# Photochemistry of Receptor-Bound Flavin Resolved in Living Human Cells by Infrared Spectroscopy

**DOI:** 10.1021/jacs.4c17815

**Published:** 2025-03-07

**Authors:** Lukas Goett-Zink, Lennard Karsten, Charlotte Mann, Hendrik Horstmeier, Jonas Spang, Kristian M. Müller, Tilman Kottke

**Affiliations:** † Biophysical Chemistry and Diagnostics, Faculty of Chemistry, Bielefeld University, Bielefeld 33615, Germany; ‡ Biophysical Chemistry and Diagnostics, Medical School OWL, Bielefeld University, Bielefeld 33615, Germany; § Cellular and Molecular Biotechnology, Faculty of Technology, Bielefeld University, Bielefeld 33615, Germany

## Abstract

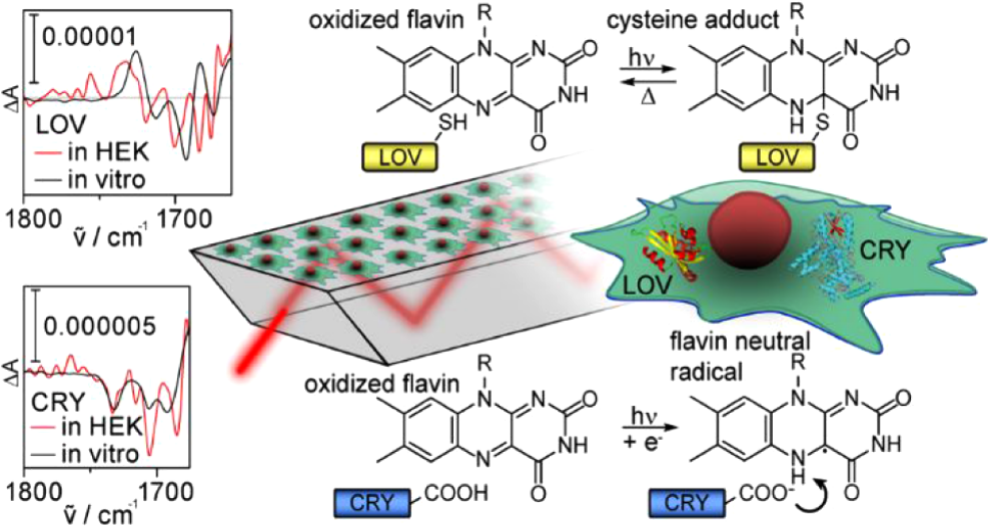

In-cell experiments on proteins have revealed that the cellular environment can exert a considerable influence on protein mechanism and structure. Here, we introduce in-cell infrared difference spectroscopy (ICIRD) as a method to study soluble receptors in living human embryonic kidney cells by applying the attenuated total reflection approach. We demonstrate on the sensory domains of plant cryptochrome and aureochrome1a, a light, oxygen, or voltage (LOV) protein, that experiments can be performed using stable and transient transfection. Cells were cultivated and transfected on an internal reflection element directly inside the spectrometer, while their viability and growth were monitored *in situ* by infrared spectroscopy. Using ICIRD, we then resolved the photoreactions of oxidized flavin to the flavin neutral radical in cryptochrome and to the flavin–cysteine adduct in LOV inside eukaryotic cells, to our knowledge for the first time, and thus confirmed their photochemical mechanisms in living human cells. However, we observed for LOV a significant upshift in signals of the carbonyl stretching modes of oxidized flavin and cysteine adduct compared to *in vitro* measurements, which could not be rationalized by effects of molecular crowding, dehydration, or temperature. Accordingly, we identified a strong impact of the eukaryotic cellular environment on the hydrogen bonding network and structure of flavin in LOV, which needs to be considered in physiology and optogenetic applications. In conclusion, we introduce ICIRD as a noninvasive, label-free approach to study soluble photoactivatable receptors in mammalian cells and provide insight into the in-cell mechanisms of two photoreceptors.

## Introduction

Light sensing is of central importance to organisms from all kingdoms of life to guide their interaction with the environment. Nature has therefore evolved a wide variety of photoreceptors for an adaptation to the light conditions. Photoreceptors sense intensity and frequency of light based on the specific cofactor and transmit this photochemical information via intracellular signaling to initiate an appropriate response by the organisms. These fascinating properties of photoreceptors have been exploited to precisely control enzymes, gene transcription, or neurons by means of optogenetics. Blue and UV-A light is sensed by the flavin-binding cryptochromes and light, oxygen, voltage (LOV) proteins, among others, which are commonly used as optogenetic tools.^1−3^

**Figure 1:**
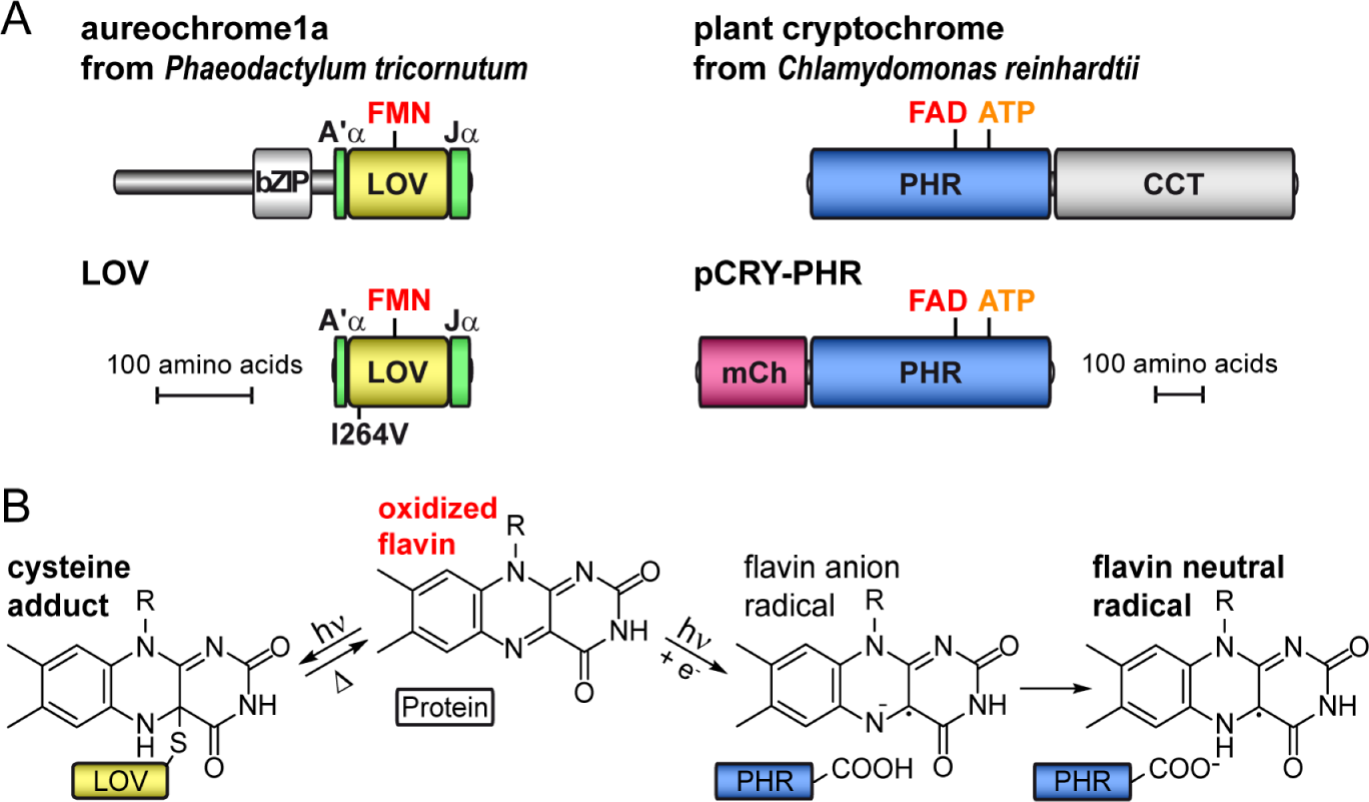
Topology and flavin-based photochemistry of aureochrome and plant cryptochrome. (A) Aureochromes consist of a DNA-binding bZIP effector and a LOV sensor domain binding FMN. Here, the LOV sensor with a point mutation I264V was investigated (LOV). Plant cryptochromes possess a CCT effector and a PHR sensor with FAD and ATP bound. In this study, mCherry (mCh) was fused to the PHR domain (pCRY-PHR). (B) Excitation of flavin with blue light leads to different photoreactions in aureochrome and plant cryptochrome. A covalent cysteine adduct with flavin is formed in aureochrome, whereas a flavin neutral radical is produced in plant cryptochrome via a decoupled electron and proton transfer.

LOV proteins have been identified in various organisms such as plants, algae, fungi or bacteria.^4^ Aureochromes are part of the LOV protein family and regulate as light-dependent transcription factors the cell division and photomorphogenesis in algae.^5,6^ The LOV domain of aureochrome noncovalently binds flavin mononucleotide (FMN) in the oxidized state as a cofactor (Figure 1A). Illumination with blue or UV-A light leads to the formation of a photoadduct with a covalent bond of cysteine to FMN and switches LOV from the dark to the light state (Figure 1B).^7,8^ Resulting conformational changes of LOV involve the hydrogen bonding network around the cofactor, a partial unfolding of two flanking α-helices and formation or rearrangement of the LOV dimer.^9−12^ These structural changes activate the effector domain of aureochromes, the basic-region leucine zipper (bZIP) and increase its affinity for target DNA.^6,9,13^

Plant cryptochromes regulate the flowering time, circadian rhythm and cell cycle in land plants and green algae.^14−16^ A highly conserved photolyase homology region (PHR) is shared among plant cryptochromes, which noncovalently binds oxidized flavin adenine dinucleotide (FAD) and adenosine triphosphate (ATP) (Figure 1A).^17^ Absorption of blue or UV-A light by plant cryptochromes results in the formation of an FAD neutral radical via a decoupled electron and proton transfer from a conserved tryptophan triad and an aspartic acid, respectively (Figure 1B).^18−20^ Subsequently, global conformational changes are initiated including α-helical elements and a rearrangement of the only β-sheet in the PHR.^21,22^ The resulting signaling state is stabilized by ATP from the milliseconds to the minute time range.^23,24^ Downstream signaling is regulated by an unstructured C-terminal extension (CCT) with low conservation.^25,26^

Photochemical and biophysical studies on photoreceptors are usually conducted *in vitro*, which substantially differs from their native environment or the operational environment in optogenetics. However, the high content of proteins and the presence of nucleotides in cells has been demonstrated to have a strong influence on the mechanisms of LOV^27^ and plant cryptochrome,^23,28^ respectively.

The structural and functional investigation of proteins in their native environment by in-cell experiments is therefore an important step toward a comprehensive understanding of their natural mechanisms. The intracellular environment including metabolites, high concentrations of macromolecules and specific binding partners cannot always be emulated by *in vitro* experiments.^29^ For instance, the structural folding and integrity of a protein can even vary between bacterial and mammalian cells depending on the protein sequence and its complex interactions with the cellular environment.^30^ Structural biology has made substantial progress in the study of proteins in various cell types including bacterial, insect, *Xenopus* or human origin.^31,32^ In particular, nuclear magnetic resonance (NMR)^32−34^ and electron paramagnetic resonance (EPR) spectroscopy^31,35^ pioneered the resolution of protein structures inside cells. A major challenge of in-cell experiments is the low concentration of target proteins in cells and the differentiation from other cellular components. Therefore, isotope, fluorescence or spin labeling has been compulsory, which is associated with a high level of experimental effort.

To overcome this limitation in the case of soluble photoreceptors, we established in-cell infrared difference spectroscopy (ICIRD) to study their photoreaction and conformational response inside living bacteria by FTIR spectroscopy,^27,36^ and further developed this method to achieve a temporal resolution of 7.6 ms.^37^ Recently, the use of quantum cascade lasers even allowed for a ns time resolution in a study on a light-driven transmembrane chloride pump.^38^ The FTIR differences induced by gas exchange were resolved for the catalytic center of hydrogenases.^39^ However, all these studies have been limited to bacterial cells and might be insufficient to cover effects by the cellular environment of eukaryotes. Rhodopsin was investigated in native animal rod cells by synchrotron FTIR spectromicroscopy, resolving tentative light-induced changes of retinal.^40^ Moreover, binding of synthetic porphyrins to proteins was demonstrated inside fixed HeLa cells using time-resolved infrared spectroscopy.^41^ To our knowledge, ICIRD on recombinant proteins in eukaryotic cells has not yet been reported.

Here, we present a method to study soluble photoreceptors in stably and transiently transfected living human embryonic kidney (HEK) cells with ICIRD using the attenuated total reflection (ATR) approach. We investigated two eukaryotic receptors, the PHR of plant cryptochrome from the green alga *Chlamydomonas reinhardtii* and the LOV domain of aureochrome1a from the diatom *Phaeodactylum tricornutum*. We verified their photochemical reactions inside human cells and found for LOV significant differences in flavin structure and hydrogen bonding between human cells, bacterial cells and *in vitro* conditions.

## Results

### Cultivation of Human Cell Lines inside an FTIR Spectrometer

**Figure 2:**
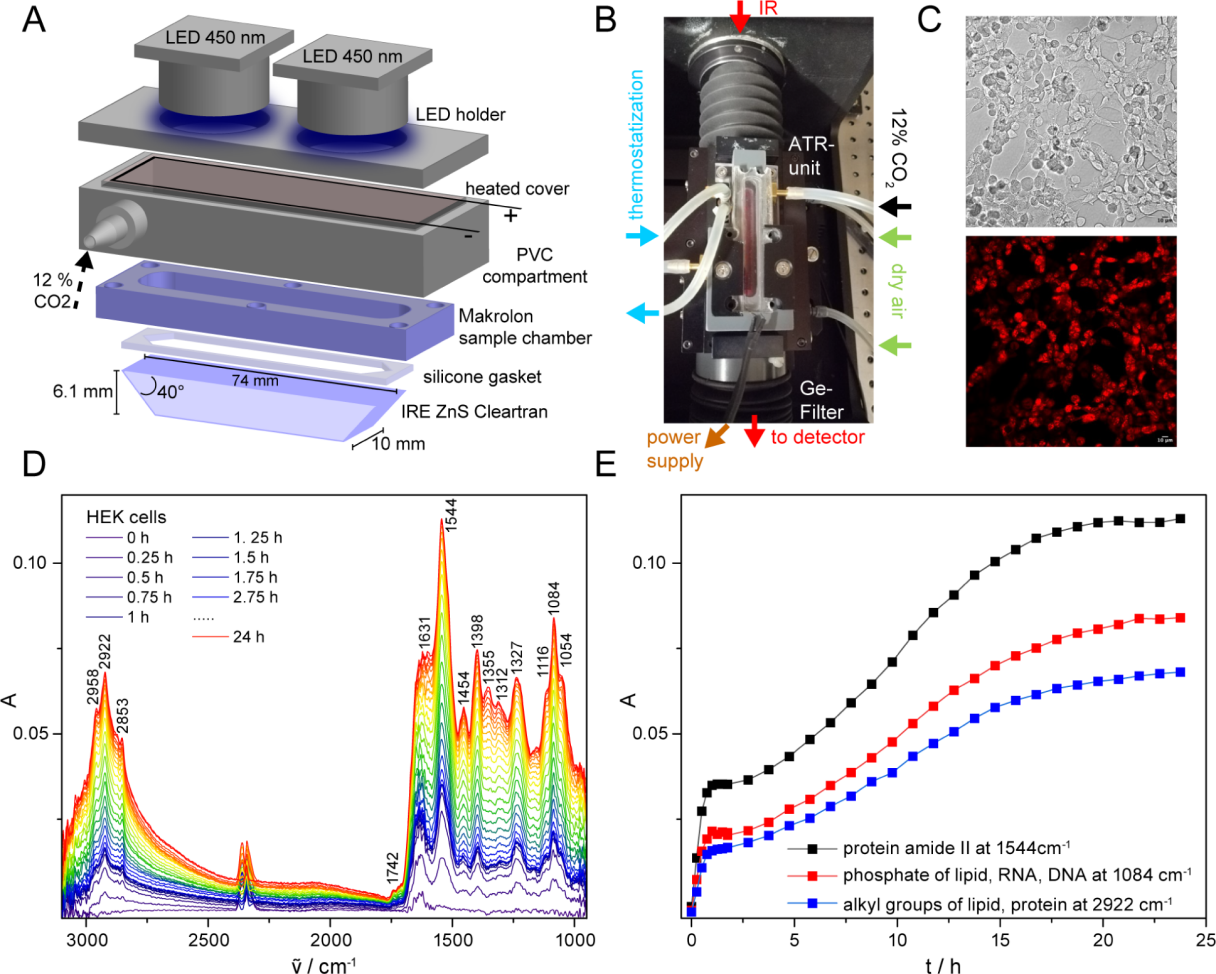
Setup for controlled cultivation of human embryonic kidney cells for in-cell infrared difference spectroscopy on soluble receptors using the ATR approach. (A) The miniaturized cultivation chamber is composed of a ZnS IRE for infrared light, a silicon gasket, a Makrolon sample chamber and a PVC compartment with a heated cover. The chamber is supplied with 12% CO_2_. Two blue LEDs are mounted at the top for illumination. (B) The cultivation chamber is built in a FTIR spectrometer and thermostatized at 37 °C. (C) Expression of pCRY-PHR in HEK cells on the ZnS IRE was verified via bright-field and fluorescence confocal imaging (ex. 561 nm, em. 600–647 nm). (D) Signals of the cellular components in the absorption spectrum were rising after seeding HEK cells on the IRE. (E) Cells started growing on the IRE after a short lag phase of 3 h dominated by sedimentation. They reached the stationary phase after 24 h. The cell growth was observed by an increase in signals attributed to proteins, lipids, DNA and RNA. Spectra were not corrected for the wavelength dependence of the penetration depth.

The investigation of human cell cultures using the ATR approach requires that the cells adhere to the internal reflection element (IRE). In addition, the penetration depth of the evanescent wave must be very large to penetrate the cytosol of eukaryotic cells for studying soluble proteins. Zinc sulfide (ZnS) was selected as material for the IRE,^42^ because it has been demonstrated to be nontoxic to human cells^43^ and its refractive index of *n*_ZnS_ = 2.23 results in a theoretical penetration depth of *d*_p_ = 1.1 μm with an angle of incidence of 40° and a refractive index of cells of *n*_cells_ = 1.37 at 1650 cm^–1^ (eq S1). The effective penetration depth of the IRE per reflection was determined for water at 1650 cm^–1^ to *d*_e_ = 1.7 μm per reflection, which is sufficient to detect cytosolic components. To cultivate human cells inside the spectrometer, a miniaturized cell cultivation chamber was developed for CO_2_ supplementation by carefully selecting materials compatible with cell growth (Figure 2A). The whole cell cultivation chamber was placed onto an ATR unit and mounted inside an FTIR spectrometer (Figure 2B). The ATR unit was tempered to 37 °C and purged with dry air.

The cultivation of HEK cells inside the spectrometer was first verified visually by bright-field microscopy (Figure S1). HEK cells cultivated on an ZnS IRE in the spectrometer or in an incubator showed similar morphology to cells in a culture flask, but were slightly more spread out on ZnS. The growth and viability of the cells in the spectrometer can be monitored *in situ* by ATR–FTIR absorbance spectroscopy. HEK cells cultivated in the miniaturized chamber showed increasing signals of cellular components over time (Figure 2D). Specific signals of lipids, proteins, DNA and RNA were detected.^44^ The amide I band at 1631 cm^–1^ showed less intensity compared to the amide II band at 1544 cm^–1^, which is caused by the displacement of water absorbing at 1650 cm^–1^ by cells at the IRE surface. The analysis of selected signals of the cellular components during incubation of the cells revealed a phase of cell sedimentation on the IRE after seeding followed by a phase of cell growth (Figure 2E). After 24 h, the cells entered a stationary phase. By monitoring the growth curve, viability of the cells can be ensured directly inside the spectrometer and without any imaging. Unhealthy cells can be identified by detachment of cells from the IRE and thus by the decrease in absorption of characteristic signals of cellular components (Figure S2).

### Aspartic Acid Deprotonates in the Photoreaction of Plant Cryptochrome in HEK Cells

For ICIRD spectroscopy on the plant cryptochrome pCRY, a stable cell line of HEK cells was generated. The fluorescent protein mCherry was fused to the PHR for selection of high-producing cell clones via fluorescence activated cell sorting (Figure S3). The expression of the resulting fusion protein pCRY-PHR (Figure 1A) in HEK cells on the IRE was first verified by confocal microscopy (Figures 2C and S4). Then, stably transfected HEK cells expressing pCRY-PHR were seeded on the IRE and cultivated inside the spectrometer.

**Figure 3:**
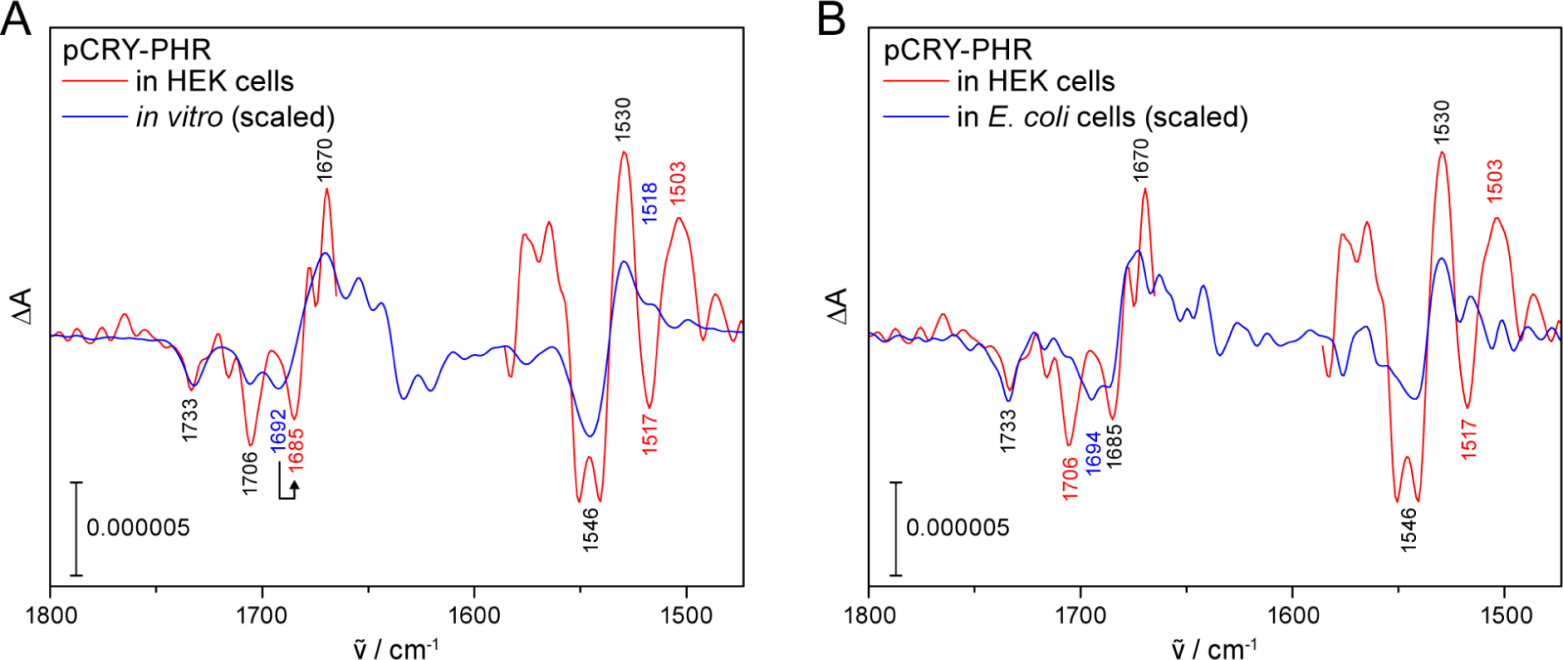
Light-induced FTIR difference spectrum of pCRY-PHR in stably transfected HEK cells compared to spectra *in vitro* and in *E. coli* cells. (A) In HEK cells, characteristic signals of the light-induced response of plant cryptochrome are detected as *in vitro* such as deprotonation of Asp396 at 1733 (−) cm^–1^ and the conversion of oxidized flavin to flavin neutral radical at 1706 (−), 1546 (−) and 1530 (+) cm^–1^. (B) The signals of the conversion of oxidized flavin are similar in HEK and *E. coli* cells including the shift to 1685 (−) cm^–1^ but differ at 1706/1694 (−) and 1517 (−)/1503 (+) cm^–1^. HEK cells were investigated with the ATR approach. 49152 scans were recorded on 26 independent preparations. The spectrum was corrected for the wavelength dependence of the penetration depth for comparison with the *E. coli* experiments in transmission configuration.

After 24 h of growth, intensity spectra were recorded before and after illumination of the cells to obtain light-induced difference spectra. Only after extensive averaging very small signals in the range of 10^–6^ in the difference spectrum were detected (Figure 3A). Absorption of water and cellular proteins at 1650 cm^–1^ caused strong noise preventing an evaluation of the amide I region at 1660–1600 cm^–1^. Nevertheless, we achieved to resolve specific signals at 1733 (−) cm^–1^ assigned to deprotonation of aspartic acid Asp396^21,23^ and at 1706 (−) cm^–1^ assigned to conversion of oxidized flavin in pCRY-PHR.^45,46^ This is, to our knowledge, the first time that the deprotonation of Asp396 in a PHR has been resolved and verified in eukaryotic cells. The reaction represents a key step in the response of plant cryptochromes to light. Additional signals of the photoreaction of the oxidized flavin to the flavin neutral radical in pCRY-PHR were observed at the same position as *in vitro* at 1546 (−)/1530 (+) cm^–1^.

The band splitting at 1546 cm^–1^ and two additional signals at 1517 (−)/1503 (+) cm^–1^ are tentatively assigned to contributions by shifted amide II signals of changes in secondary structure in the HEK cells. This assignment needs to be confirmed by future analysis of the amide I region.

To check whether differences in the signals are specific to HEK cells or represent a general effect of cellular components, we studied the response of pCRY-PHR in bacterial cells (Figure 3B). For both, HEK cells and *E. coli*, the C(4)=O stretching mode of flavin was downshifted to 1685 (−) cm^–1^ pointing to a general effect. The other C(4)=O stretch signal at 1706 (−) cm^–1^ is highly variable in intensity and position in agreement with previous findings on PHR and full length pCRY.^36^ This comparison indicates that the response of pCRY-PHR in *E. coli* resembles that in HEK cells in general, but might deviate at specific signals originating from the flavin and secondary structure.

### Formation of the Flavin Adduct of LOV in HEK Cells

**Figure 4:**
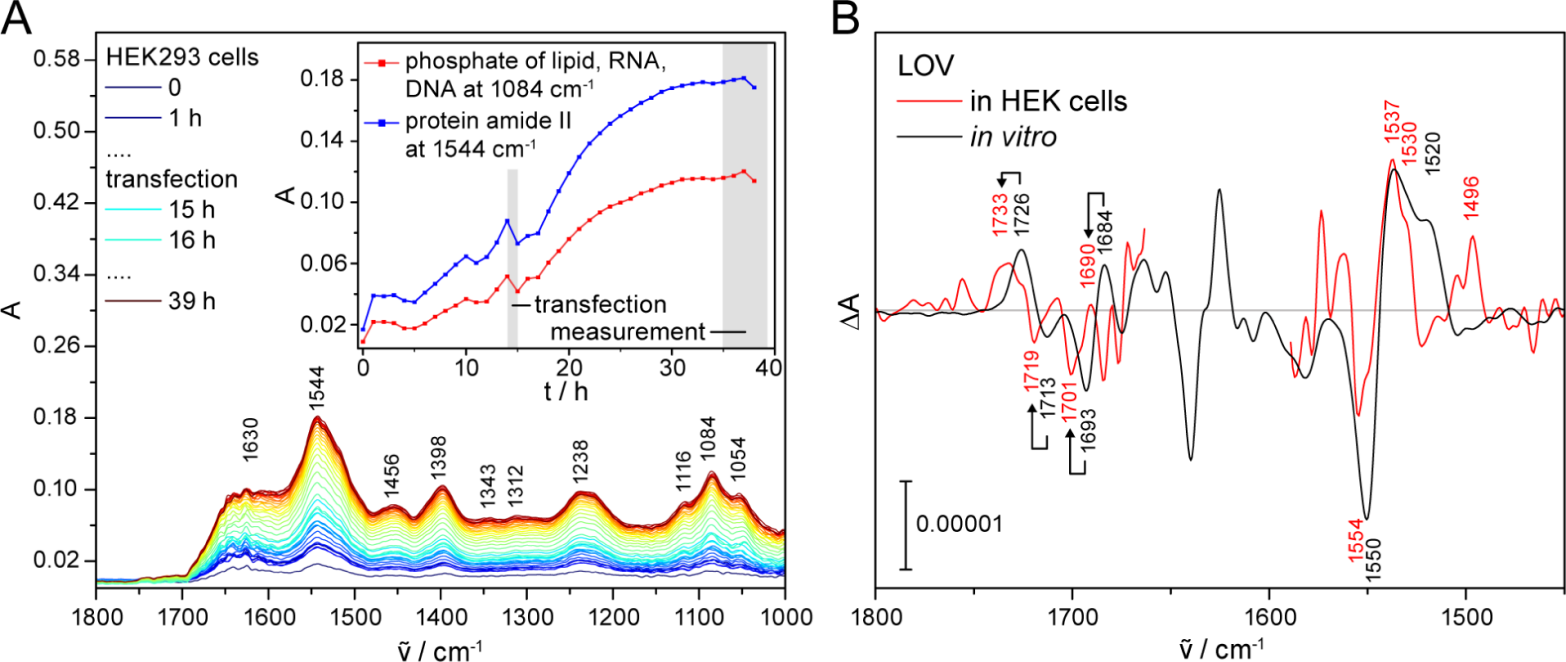
Cell growth of transiently transfected HEK cells as monitored by ATR–FTIR spectroscopy *in situ* and light-induced FTIR difference spectrum of LOV in living HEK cells. (A) The characteristic signals for the cellular compounds increase in intensity before and after transfection reaching a limit at 32 h upon which the measurement was started (inlet). (B) The light-induced formation of the flavin adduct and the loss of oxidized flavin are evidenced in the difference spectrum of LOV in HEK cells by signals at 1733 (+), 1719 (−), 1701 (−) and 1690 (+) cm^–1^. In the region at around 1550 cm^–1^, difference signals originate from flavin adduct formation and amide II vibrations caused by secondary structural changes of the protein moiety. Of note, all signals in the region of 1740–1680 cm^–1^ are shifted to higher wavenumber by the HEK cell environment compared to *in vitro* as indicated by arrows. 8448 scans were recorded on 3 independent preparations. Spectra were corrected for the wavelength dependence of the penetration depth, but some distortion in intensity by anomalous dispersion remains at 1600–1500 cm^–1^.

Next, we aimed to verify if deviations in the flavin response in HEK cells compared to *in vitro* are specific for plant cryptochrome. We hence focused on a different blue light receptor, the LOV sensor of aureochrome1a from *Phaeodactylum tricornutum*. Developing an additional stable cell line for LOV is accompanied by a high experimental and temporal effort. Therefore, we established a protocol for the transient transfection of HEK cells on the IRE directly inside the FTIR spectrometer. HEK cells cultivated inside the spectrometer were transfected with a plasmid for expression of LOV 14–15 h after seeding. The I264V point mutation accelerates the recovery of the dark form by a factor of up to 20 compared to the wild type^47^ (Figure S5) without altering the mechanism (Figure S6A), allowing for multiple measurements on transfected cells and thus reducing the experimental effort. The viability of the cells was monitored by absorbance spectra up to 39 h (Figure 4A). After transfection, cells showed reduced viability and growth for 3 h followed by strong cell growth reaching a limit at ∼32 h (Figure 4A). A temporary loss of viability after transfection is consistent with a known minor cytotoxicity of the transfection reagent. Approximately 35 h after seeding, the cells were illuminated and difference spectra were recorded.

Signals of the photoreaction of LOV in HEK cells were observed in the light-induced ICIRD spectrum that are approximately six times stronger than in the spectrum of pCRY-PHR in HEK cells (Figures 3A and 4B), which might be because of a higher expression level of LOV holoprotein in the transiently transfected cells compared to stably transfected cells. Accordingly, transient transfection is a suitable approach for ICIRD on human cell lines. The characteristic carbonyl signals of the formation of the cysteine adduct from the oxidized flavin^45−47^ were resolved at 1733 (+), 1719 (−), 1701 (−) and 1690 (+) cm^–1^ (Figure 4B). In the region from 1570 to 1500 cm^–1^, the spectrum showed bands at 1554 (−) and 1537 (+) cm^–1^ originating from ring vibrations of the flavin photoconversion to the adduct and overlapping with amide II vibrations from secondary structural changes.^45−49^ The absorption of water impeded the evaluation in the spectral range of amide I from 1660 to 1600 cm^–1^. In the region between 1450 and 1200 cm^–1^, we observed a shift of all signals to lower wavenumbers, attributed to anomalous dispersion in the ATR measurements caused by the lower refractive index of ZnS compared to common IRE substrates and by measurements near the critical angle of incident for total reflection (Figure S7).^50,51^ To our knowledge, the formation of the cysteine adduct in eukaryotic cells has been demonstrated here for the first time. Further evidence may be provided by resolving the SH vibration of cysteine at around 2570 cm^–1^.^38,45,52^

Comparing the spectrum of LOV in HEK cells with *in vitro*, it is evident that the carbonyl signals at 1733 (+), 1719 (−), 1701 (−) and 1690 (+) cm^–1^ are shifted approximately 7 cm^–1^ to higher wavenumbers compared to the signals *in vitro* at 1726 (+), 1713 (−), 1693 (−) and 1684 (+) cm^–1^ (Figure 4B). Likewise, a minor shift to higher wavenumbers at 1554 (−) and 1537 (+) cm^–1^ can be detected. A similar shift of signals to higher wavenumbers was not observed in the spectrum of pCRY-PHR in HEK cells (Figures 3A and 4B). This shift accordingly cannot be justified by any anomalous dispersion of the ATR approach and has not been reported previously. To exclude other causes, we recorded spectra of LOV in *E. coli* cells (Figure S8), *in vitro* on the same IRE (Figure S9) and *in vitro* at 37 °C (Figure S6B), which all are identical in this spectral region. Moreover, an additional and specific signal in HEK cells is observed at 1496 (+) cm^–1^, which we tentatively assign to a shifted amide II contribution from 1520 cm^–1^*in vitro* originating mainly from β-sheet changes in LOV.^37^ Therefore, we conclude that differences in signals at 1740–1680 cm^–1^ and 1554–1490 cm^–1^ compared to *in vitro* originate specifically from the cellular environment of HEK cells, affecting LOV and its flavin cofactor.

## Discussion

### ICIRD to Study Photochemistry and Structural Response of Soluble Proteins in Mammalian Cells

The influences of the cellular environment on protein mechanisms are mostly neglected by biophysical characterizations, in part because of a lack of suitable techniques. Here we report on a novel spectroscopic approach for the investigation of soluble proteins in living human cell lines. ICIRD can be applied to either transiently or stably transfected cells and so far, provides insight into the structure, photoreaction and chemical environment of the cofactor in receptors, as demonstrated on two different types of blue light receptors, plant cryptochrome and LOV protein. These receptors occur naturally in eukaryotes such as plants, fungi and algae, whereas HEK cells do not express LOV or plant cryptochrome. Accordingly, we studied the receptors in a system without any natural background of expression. A contribution by inherent human cryptochromes can be excluded by the detected deprotonation of Asp396 exclusively found in plant cryptochromes. HEK cells are a widely accepted model system for studying proteins from eukaryotes such as plants and provide a representative cellular environment for eukaryotes. In addition, utilization of optogenetic tools in human cell lines is well established including several tools based on LOV and plant cryptochrome.^2,3^ The characterization of these tools in HEK cells is important to understand their function and mechanism in the operational environment. The investigation of receptors in their native host cells by ICIRD is a considerable challenge for future experiments, because these cells express a variety of (flavin-binding) receptors that respond to light. The contributions of the specific receptor will only be isolated by a study on strongly overexpressing and knockout strains in direct comparison.

A major challenge for in-cell spectroscopy on human cell lines is to ensure the cell viability. The cellular metabolic state can influence the protein structure, as it was observed in healthy and stressed cells.^53^ Therefore, flow bioreactors have been implemented for in-cell NMR experiments, allowing for a cultivation of HeLa cells up to 24 h.^54,55^ In our approach, HEK cells were similarly cultivated inside the spectrometer for over 35 h by using the ATR technique. Moreover, the cell growth as well as the viability can be monitored *in situ* ensuring measurements on living cells. The cells are accessible during the cultivation and can even be transfected directly inside the spectrometer. Major advantages of using transiently transfected cells over stable cell lines are the lower experimental effort and the fact that no fluorescence reporter is required.

### Photoreaction of Flavin in Plant Cryptochrome and LOV Proteins in a Eukaryotic Cellular Environment

The presence of the flavin neutral radical as photoproduct in plant cryptochromes after illumination has been demonstrated previously in insect cells by EPR spectroscopy and in bacterial cells using EPR, FTIR, and UV–vis spectroscopy.^28,36,56^ Here, the insight was significantly extended by detecting the deprotonation of Asp396 in the photoreaction in a eukaryotic cell, confirming this proton transfer to flavin as a key step in the light response of plant cryptochromes for a plant-like cellular environment.

**Figure 5:**
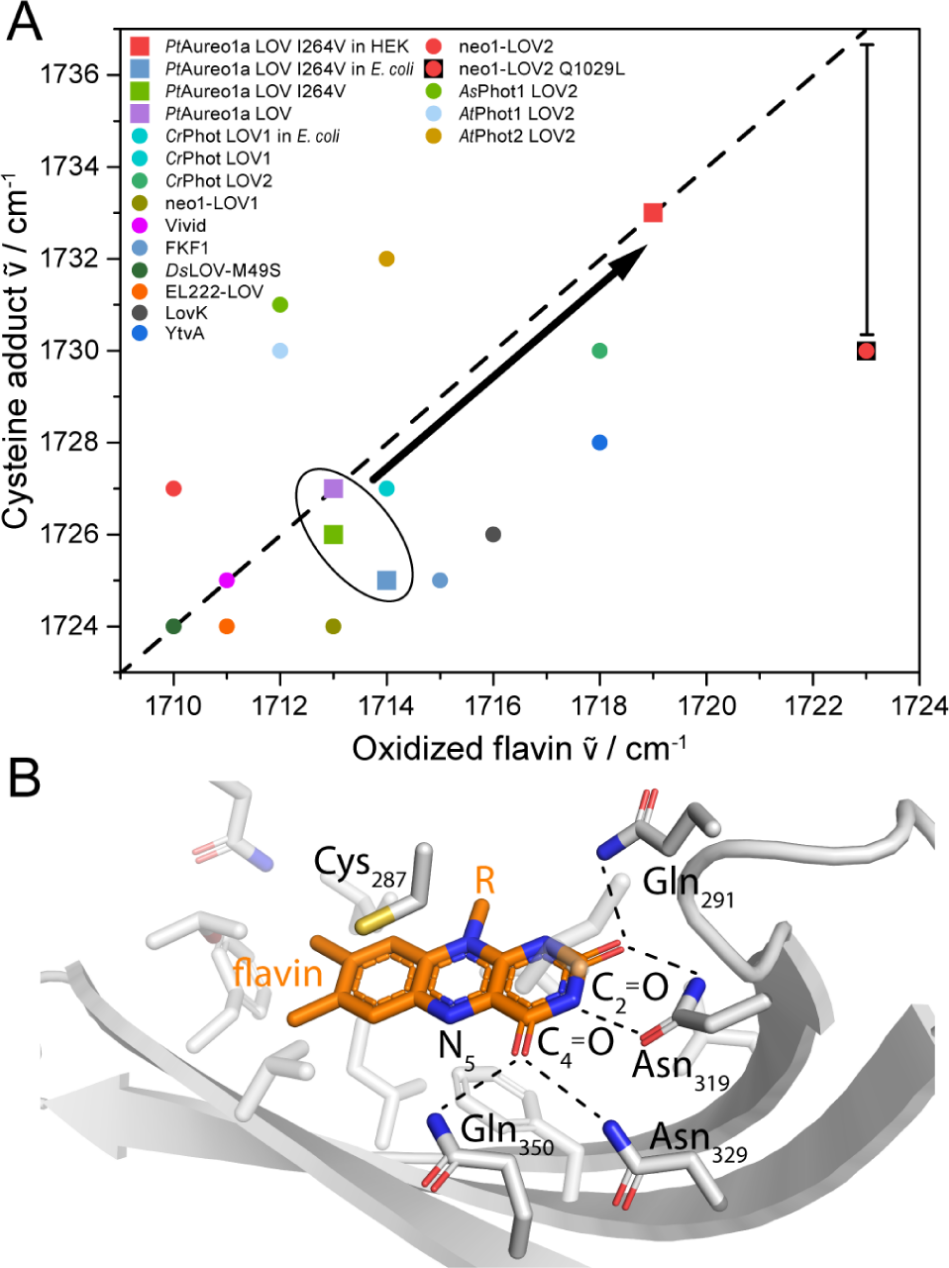
Comparison of the carbonyl signals of flavin in different LOV domains in the dark and light state and structural environment of flavin in LOV. (A) The signals of oxidized flavin and the cysteine adduct of LOV in HEK cells are upshifted by ∼7 cm^–1^ (black arrow) compared to *in vitro* and occur at higher wavenumbers than in any other LOV domain. The difference in frequency between the cysteine adduct and the oxidized flavin is usually ∼14 cm^–1^ (dashed line). The nonfunctional mutant Gln1029Leu of neo1-LOV2 does not follow this behavior as indicated by the vertical bar. Spectral positions of the flavin signals and references are listed in Table S1. (B) In LOV, Gln291, Asn319, Asn329 and Gln350 form hydrogen bonds to C(2)=O and C(4)=O of flavin (PDB entry 5A8B). Upon illumination, Cys287 reacts with the flavin resulting in a flip of Gln350 and a new hydrogen bond with N(5)–H.

LOV proteins in bacterial cells have already been studied by fluorescence spectroscopy demonstrating an impact of the cellular environment on the kinetics of the dark state recovery.^57^ With fluorescence spectroscopy, only the presence of oxidized flavin can be detected through its characteristic emission spectrum, whereas the adduct is nonfluorescent. In addition, infrared spectroscopy provides structural information about the conversion of oxidized flavin into the cysteine adduct in HEK cells via the stretching modes of flavin. Based on our results, we confirm that the generally accepted photoreaction of LOV proteins also takes place in HEK cells. However, carbonyl signals of the cysteine adduct and oxidized flavin in LOV are shifted to higher wavenumbers by 7 cm^–1^ in HEK cells compared to *in vitro* (Figure 5A). A comparison with other LOV domains shows that the signals in HEK cells are at higher wavenumbers than those of any other domain characterized previously including results from experiments in living *E. coli* cells (Figure 5A, Table S1). Only the LOV1-C57S-LOV2 tandem domain of phototropin from *C. reinhardtii* (*Cr*Phot LOV2) shows already *in vitro* high wavenumbers in the comparison but is positioned clearly lower than LOV in HEK cells. LOV-effector proteins have been excluded because they did not show any difference in signal position to the isolated LOV domains.

A shift to higher wavenumbers can be explained by a decrease in hydrogen bonding to the carbonyls of flavin in LOV and might be rationalized either by changes in the orientation of amino acids in the cofactor binding pocket or a shielding of flavin from the bulk polar environment. Binding of intracellular proteins or small molecules only present in eukaryotic cells might cause these changes of LOV in HEK cells. Posttranslational modifications are less likely to affect the carbonyl moiety of flavin, as the predicted modification sites (see Supporting Information) are located at the protein surface and are distant from the cofactor. Molecular crowding is excluded as an explanation because this should be similarly effective in *E. coli*. Strong dehydration would result in an opposite shift of the cysteine adduct signal to lower wavenumbers caused by suppressed conformational changes.^58,59^ Any temperature effects can be excluded as the two signals are insensitive to changes in temperature between 10 and 37 °C (Figure S6B). An influence of the temperature on the signal of the cysteine adduct was reported, but at below −20 °C.^46,58,59^ Interestingly, the reported sensitivity of LOV domains to temperature and hydration only affects the cysteine adduct,^58,59^ whereas the influence of the cellular environment impacts the carbonyls of flavin in both the light state and dark state.

A strong upshift in signals of the oxidized flavin of 13 cm^–1^ compared to the wild type was reported in a Gln1029Leu mutant of neo1-LOV2 (Figure 5A).^60^ In wild-type LOV, this conserved glutamine (Gln350) is hydrogen-bonded to the C(4)=O in the dark (Figure 5B). Accordingly, the lacking H-bond and the hydrophobic leucine cause the upshift of 13 cm^–1^ in signal in the mutant. However, this does not explain why in the Glu1029Leu mutant the shift of the cysteine adduct is only 3 cm^–1^ compared to wild type. The difference in wavenumbers between the oxidized flavin and the cysteine adduct in various LOV domains ranges from 10 to 19 cm^–1^ and is typically ∼14 cm^–1^ (dashed line in Figure 5A). Small deviations from 14 cm^–1^ can be nicely explained by “vibrational phylogeny”, as LOV of bacterial origin are found below the line and all homologues of eukaryotic phototropin-LOV2 are above the line. The nonfunctional Gln1029Leu clearly deviates from this pattern with a difference in signals of only 7 cm^–1^. This difference mainly reflects the adduct formation to the flavin C(4a), which contributes +6 cm^–1^ according to DFT calculations.^61^ The full 14 cm^–1^ shift therefore is caused by the flip of the glutamine side chain to accept a hydrogen bond from of the flavin upon illumination and concomitant conformational changes.^60^ LOV in HEK cells is consistent with the typical observation of 14 cm^–1^ (Figure 5A). Accordingly, we consider LOV in HEK cells to be fully functional including the glutamine flip taking place upon illumination. This flip represents a key element in signaling of most LOV domains.^60,62^

In LOV, Gln291, Asn329 and Asn319 form hydrogen bonds to C(2)=O and C(4)=O of the flavin, respectively, in both dark and light states (Figure 5B).^9^ A possible scenario for the 7 cm^–1^ upshift in HEK cells involves a disruption of hydrogen bonding between Asn329 and flavin along with a decrease in interaction of Asn319 with flavin, which would result in an upshift of 10 cm^–1^ for the C(4)=O signal at 1713 cm^–1^, as demonstrated by DFT calculations (Figure S10C). However, the calculated IR spectra do not explain the additional signals at 1693 and 1701 cm^–1^ for *in vitro* and in cell experiments, respectively. The second signal has previously also been assigned to C(4)=O, caused by a heterogeneity of the protein moiety depending on whether or not Asn329 forms a hydrogen bond to C(4)=O.^59^ As an alternative scenario, coupling of C(4)=O with the surrounding amino acids residues might result in a high and low frequency component, which is supported by DFT calculations including such coupling (Figure S10F and Table S2). The calculated shift by rotating Asn329 is only +3 cm^–1^ for both signals and cannot fully explain the experimentally observed shift for both signals of +7 cm^–1^. Accordingly, we propose a rearrangement of Asn329 and Asn319, which might be caused by binding of HEK-specific proteins to the β-sheet surface of LOV at which the asparagine residues are located. In different LOV domains this β-sheet surface regulates and interacts with effectors,^63^ which is commonly utilized in optogenetics. Therefore, our results indicate that HEK-specific proteins may compete with LOV effectors and the flanking helices of LOV for binding to the β-sheet, impacting the functionality of LOV-based sensors and optogenetic tools.

In summary, the cellular environment of eukaryotic cells has a strong impact on LOV domains in both, the dark and light state, by an interaction with small molecules or proteins causing a change in LOV structure reflected by the loss of hydrogen bonds to flavin. These binding partners await identification.

## Conclusions

The emerging challenge of studying mechanisms of receptors in their natural environment was addressed in this work by developing an infrared spectroscopic approach, ICIRD, on human cell lines. The combination of a miniature cultivation chamber and the ATR approach allowed us to cultivate HEK cells and monitor their growth and viability *in situ* inside an FTIR spectrometer. ICIRD is a noninvasive technique that does not require the use of D_2_O medium, isotope or spin labeling. However, our approach is limited to adherent cells by the inherent low penetration depth. The investigation of mechanisms of receptors by ICIRD requires an overexpression, since the response of the receptor needs to be resolved against an enormous cellular background by the proteome. The discrimination is ensured by the inherent ability of difference spectroscopy to select only for light-induced changes. At low expression levels of the target protein, also other endogenous, light-sensitive reactions might contribute to the signal. For now, ICIRD has only been applied to photoreceptors, but might be expanded to nonphotosensitive proteins by activating them with established optogenetic and photopharmacological approaches.^1,64^ Low concentration of protein and strong absorbance by H_2_O impeded the evaluation of the amide I region, in which changes in secondary structure are found. The implementation of quantum cascade lasers as probe light might provide access to this important region and might even allow for time-resolved investigations of (secondary) structural changes of proteins in eukaryotic cells.

By stable or transient transfection with genes encoding for LOV and cryptochrome-PHR, we were able to investigate their flavin-based photoreaction in human cell lines. Based on our results, we confirm that the generally accepted photoreactions of both, LOV and plant cryptochromes, take also place in eukaryotic cells. However, for LOV we detected shifts in signals of the flavin cofactor in the dark and light state, which reflect a specific impact of the eukaryotic cellular environment on the photoreceptor attributed to binding of small molecules or proteins. The carbonyl stretching modes of flavin serve as a probe for the structure and hydrogen-bonding network of the cofactor in the photoreceptor and therefore for the structure of flavin and protein moiety. Hence, the shift in signals of the flavin cofactor may represent an indicator for the differences induced by the cellular environment including protein–protein interactions, kinetics, and catalytic activity. Such properties are not only important for the application in optogenetic tools but also for the function of these photoreceptors in their natural environment.

## Supporting Information Available

Experimental procedures, seeding of HEK cells on ZnS IRE (Figure S1), cell growth on IRE inside the spectrometer (Figure S2), cell sorting experiments using FACS (Figure S3), confocal images of HEK cells on IRE (Figure S4), dark state recovery of LOV (Figure S5), FTIR difference spectra of LOV wild type and mutant at different temperatures (Figure S6), corrected and uncorrected ICIRD spectra of LOV in HEK cells (Figure S7), FTIR difference spectra of LOV in bacterial cells and *in vitro* (Figure S8), FTIR difference spectra of LOV *in vitro* recorded with the ATR approach and in transmission mode (Figure S9), calculated FTIR spectra of flavin in LOV using DFT (Figure S10 and Table S2), and spectral positions of carbonyl signals of flavin in different LOV domains (Table S1) (PDF)

## Abbreviations

LOV: light, oxygen, or voltage
FMN: flavin mononucleotide
bZIP: basic-region leucine zipper
PHR: photolyase homology region
FAD: flavin adenine dinucleotide
ATP: adenosine triphosphate
CCT: C-terminal extension
NMR: nuclear magnetic resonance
EPR: electron paramagnetic resonance
ICIRD: in-cell infrared difference spectroscopy
HEK: human embryonic kidney
ATR: attenuated total reflection
IRE: internal reflection element
