# Supplementary material for: Photochemistry of Receptor-Bound Flavin Resolved in Living Human Cells by Infrared Spectroscopy
Source: J Am Chem Soc. 2025 Mar 7;147(11):9676–85. doi: 10.1021/jacs.4c17815 (PMC11926855; doi:10.1021/jacs.4c17815)
Supplement: Supplementary file 1 — ja4c17815_si_001.pdf [file ja4c17815_si_001.pdf]

Supporting Information

Photochemistry of Receptor-Bound Flavin Resolved in Living Human Cells by Infrared Spectroscopy

Lukas Goett-Zink[1,2], Lennard Karsten[3], Charlotte Mann[3], Hendrik Horstmeier[1], Jonas Spang[1], Kristian M. Müller[3], and Tilman Kottke[1,2]

From the [1]Biophysical Chemistry and Diagnostics, Faculty of Chemistry, Bielefeld University, Bielefeld 33615, Germany; [2]Biophysical Chemistry and Diagnostics, Medical School OWL, Bielefeld University, Bielefeld 33615, Germany; [3]Cellular and Molecular Biotechnology, Faculty of Technology, Bielefeld University, Bielefeld 33615, Germany

For correspondence: Tilman Kottke, tilman.kottke@uni-bielefeld.de; Lukas Goett-Zink, lukas.goett-zink@uni-bielefeld.de

Experimental Procedures

Construction of Expression Vectors

For bacterial expression, the coding sequence of LOV (aa 238-378) of aureochrome1a from *Phaeodactylum tricornutum* with the mutation I264V and an N-terminal His_6-tag was synthesized and cloned into a pET28a(+) vector using NcoI and XhoI restriction sites (pET28a(+)-LOV) by Twist Bioscience HQ (San Francisco, USA).

For mammalian expression, the coding sequence of LOV (aa 238-378) of aureochrome1a from *Phaeodactylum tricornutum* with the mutation I264V was synthesized and cloned into a pTwist CMV Hygro vector using the restriction sites NotI and XbaI (pTwistCMV-LOV) by Twist Bioscience HQ (San Francisco, USA).

The expression plasmid for the hyperactive sleeping beauty transposase variant (SB100X) pCMV(CAT)T7-SB100 (Addgene #34879, SeH_P069, pZMB0794)[1] and the transposon donor plasmid pT2/BH_IR/DR (SeH_P068, pZMB0793) based on Izsvák and Ivics[2] were kindly provided by Sebastian Hanke, Molecular Biotechnology, University of Potsdam.

The expression cassette CMV-MSC-bGHpA of mammalian expression vector pcDNA5/FRT (V6010-20, Thermo Fisher Scientific, Germany) was digested with NruI and PvuII and blunt-end cloned between inverted repeats IR/DR of the transposon donor vector pZMB0793, which was linearized with MscI and EcoRV. The resulting intermediate cloning product was named pT2/BH_IR/DR-pCMV-MSC-bGHpA (pZMB0812).

The NdeI recognition site within the mCherry (mCh) coding sequence of the plasmid pHR-FUSN-mCh-Cry2WT (Addgene #101223, pZMB0756)[3] was silently mutated from CATATG into CACATG. Mutated mCh coding sequence was amplified using the overhang primers 5‘-TTTTTGCTAGCATGGTGTCTAAAGGCGAGG-3‘ and 5‘-AAAAAGCGGCCGCTCACTTGTACAATTCATCCATGCC-3‘ and cloned into the transposase donor vector pZMB0812 using the restriction enzymes NheI and NotI. The resulting intermediate cloning product was named pT2/BH_IR/DR-pCMV-mCh-bGHpA (pZMB0832).

The PHR coding sequence of pCRY from *C. reinhardtii* was amplified from the template plasmid pET11a_FL-pCRY[4] using the overhang primers 5‘-TTTTTTGTACAAGGGTGGAGGGTCCCCCACGAGTTTAAGACC-3‘ and 5‘-AAAAAGCGGCCGCTCATTTCTCGAACTGTGGATGGCTCCAGGAAGCATAACGACC GCTGCTTTCG-3‘ to genetically fuse the Strep-tag II WSHPQFEK to the C-terminal PHR. The amplicon was cloned into the transposase donor vector pZMB0832 using the restriction enzymes BsrGI and NotI. The resulting plasmid was named pT2/BH_IR/DR-pCMV-mCh-PHR-StrepII-bGHpA (pZMB0835) coding for the expression cassette of the fusion construct pCRY-PHR under the control of CMV promoter and bGH polyadenylation signal. The transposon donor plasmid containing the expression cassette flanked by the transposase binding sites IR/DR enables fairly random genomic insertion, when co-transfecting cells with the SB100X transposase encoding plasmid pZMB0794.

The mCh-PHR-StrepII insert was amplified from the plasmid template pZMB0835 using the overhang primers 5‘-TTTTTCATATGGTGTCTAAAGGCGAGG-3‘ and 5‘-CTAGACTCGAGCGGCC-3‘. The amplicon was cloned into the bacterial expression vector pET21a (+) (Addgene #69740-3) using the restriction enzymes NdeI and XhoI. The resulting

cloning product pET21a_mCh-PHR_StrepII (pZMB0837) enables for bacterial pCRY-PHR expression and protein purification via Strep-tag II capture.

Cell Culture

HEK-293 cells (ACC 305, DSMZ, Braunschweig, Germany) were cultured in *Dulbecco's modified eagle* medium - high glucose (DMEM, Sigma-Aldrich, Steinheim, Germany) supplemented with 10% (v/v) fetal calf serum (FBS Superior, Sigma-Aldrich Steinheim, Germany) and 1% (v/v) penicillin/streptomycin (10,000 U penicillin, 10 mg/mL streptomycin, Sigma-Aldrich) and incubated at 37 °C and 5% CO_2 if not mentioned otherwise.

Stable Transfection of HEK Cells Using the Sleeping Beauty Transposon System

HEK293 cells were seeded at a density of 3×10^6 cells per 100 mm dish the day before transfection. A total amount of 15 µg DNA per 100 mm dish was transfected using calcium phosphate. SB100X expressing plasmid pZMB0794 and transposon donor plasmid pZMB0835 were mixed in a 1:1 molar ratio in 500 µL $CaCl_2$ (3 M, pH 7.05) buffer. The DNA/$CaCl_2$ solution was rigorously vortexed and dropwise added to 500 µL 2×HBS (50 mM HEPES, 1.5 mM NaH_2PO_4, 280 mM NaCl, pH 7.05) buffer. The DNA/calcium phosphate solution was vortexed and dropwise added to the 100 mm cell culture dish. Cells were washed with PBS (500 mM NaCl, 100 mM KCl, 10 mM Na_2HPO_4, 10 mM KH_2PO_4, pH 7.5) buffer 48 h post-transfection and subsequently prepared for cell sorting.

Cell Sorting

After stable transfection, pCRY-PHR expressing HEK293 cells were selected for mCherry fluorescence using Bio-Rad S3e cell sorter (ex. 488/561 nm). In preparation for cell sorting, cells were trypsinized (0.25%, 2.5 g porcine trypsin, 0.2 g EDTA×4 Na per liter of Hanks' Balanced Salt Solution with phenol red, Sigma-Aldrich, Steinheim, Germany), suspended in DMEM and filtered through a 35 µm cell strainer cap. The fluorescence in FL3 channel (em. 602-627 nm) of live cells, gated for live cells in forward scatter (FSC)-area versus side scatter (SSC)-area plot, was determined in comparison to nontransfected HEK293 control cells. Live cells were gated for sorting that showed a minimum of 100× higher fluorescence intensity compared to that of the nontransfected control population in FL3 channel caused by high expression level of mCh-PHR. 0.8×10^6 events were sorted into DMEM and seeded into a T-25 cell culture flask. For maintenance, the sorting procedure was repeated every fortnight.

Microscopy

For microscopy, 1 mL with 1.5×10^6 pCRY-PHR-expressing HEK-293 cells in DMEM were seeded on a zinc sulfide internal reflection element (ZnS IRE) and incubated in the dark with an additional source of moisture (2 mL sterile H_2O in a 35 mm dish placed next to the IRE) to prevent drying. 24 h post seeding of the cells, the IRE was placed on a microscopy cover slip (24×60 mm) and HEK-293 cells were imaged using confocal fluorescence microscope (LSM 780, Zeiss, Oberkochen, Germany). The objective LD LCI Plan-Apochromat 25×/0.8 Imm Korr DIC M27, the main beam splitter (MBS) 488 / 561 nm, and a laser at 561 nm (6% power, 1.70 Airy unit) were used. Emission detection at 600 - 647 nm was performed using a photomultiplier tube. Bright-field images were acquired using a DMI6000 B inverted microscope (Leica Microsystems) with a 10×

objective to check the morphology of HEK-293 cells growing on a ZnS IRE within the miniaturized culture chamber of the FTIR spectrometer or within a commercial cell culture chamber.

Protein Expression and Purification

pCRY-PHR and LOV were expressed with a C-terminal Strep-tag in *E. coli* SoluBL21 (DE3) using a pET21a(+) vector (pZMB0387) and with an N-terminal His_6-tag in *E. coli* BL21 (DE3) using a pET28a(+) vector (pET28a(+)-LOV), respectively. The cells were incubated at 37°C and 120 rpm in DYT medium with 50 µg/mL kanamycin or 200 µg/mL ampicillin to an OD_{600} of 0.5 and subsequently cooled to 18 °C. After further cultivation at 18 °C, the expression was induced with 10 µM isopropyl-β-D-thiogalactopyranoside at an OD_{600} of 0.8. Cells were cultivated for 20 h in the dark and subsequently harvested by centrifugation (6000 ×g, 20 min, 4 °C).
Cells expressing pCRY-PHR and LOV were disrupted via French press (SLM Amincor, 1000 bar, 2 cycles) using a buffer consisting of 50 mM sodium phosphate buffer, 100 mM NaCl, 20% glycerol, pH 7.8 and 50 mM potassium phosphate buffer, 300 mM NaCl, 20 mM imidazole, pH 8, respectively, with protease inhibitor (cOmplete EDTA-free protease inhibitor cocktail, Roche, Mannheim, Germany) and DNase I. The cell lysate was centrifuged for one hour with 108,000 g at 4 °C (Avanti J-30 I, Beckman Coulter, Brea, USA) and the supernatant was applied to a Strep-Tactin-Sepharose (IBA-Lifesciences, Göttingen, Germany) column (15 mL column volume) manually packed or on a prepacked 5 mL Ni-charged IMAC (EconoFit Nuvia IMAC, BioRad, Hercules, USA) column via an FPLC system.
For pCRY-PHR, the column was incubated with the supernatant for 2 h at 4 °C, washed with 50 mM sodium phosphate buffer, 100 mM NaCl, 20% glycerol, pH 7.8 and the protein eluted with 12.5 mM D-desthiobiotin. The protein was washed with 50 mM sodium phosphate buffer, 100 mM NaCl, 1% glycerol, 5 mM ATP, pH 7.8 and concentrated using an ultrafiltration concentrator (Sartorius, Göttingen, Germany) to 1.3 mM (110 mg/mL) for FTIR difference spectroscopy.
For LOV, a linear gradient from 20 mM to 300 mM imidazole with 50 mM potassium phosphate buffer, 300 mM NaCl, pH 8 was applied at 4 °C, in which LOV eluted at 100 mM imidazole concentration. LOV was washed with 50 mM potassium phosphate buffer, 300 mM NaCl, pH 8 and reconstituted with 1.5 excess of FMN to protein over night at 4 °C. The next day, LOV was washed with buffer and concentrated to 1.7 mM (30.5 mg/mL) for FTIR difference spectroscopy.

Infrared Spectroscopy

The FTIR difference spectra of pCRY-PHR and LOV *in vitro* were recorded on a Bruker IFS66/v spectrometer. A long pass filter with a cut off at 2000 cm^{-1} or 1910 cm^{-1}, respectively, was placed in front of the mercury cadmium telluride (MCT) detector to increase the intensity of infrared light in this spectral region and thereby reduce the noise. Spectra were acquired at 10 °C with a scanner velocity of 150 kHz and a resolution of 4 cm^{-1}. For pCRY-PHR, ~1.4 µL of the protein sample (1.3 mM) was placed between two BaF_2 windows, sealed with grease and mounted inside the spectrometer. A difference spectrum was obtained by recording 1024 scans before and after illuminating the sample for 5 s with a blue LED (450 nm, 14 mW/cm^2, Lumileds, Schiphol, Netherlands). The difference spectrum of pCRY-PHR *in vitro* resulted from 5120 scans on 5 technical replicates.
For LOV measured with the ATR technique, 1.5 mL of the sample with a concentration of 1.7 mM was placed on a ZnS IRE (CRYSTAL GmbH, Berlin, Germany) with 8 active reflections and an

effective pathlength of d_e =14 µm at a 40° angle of incidence, which was mounted on an ATR unit (Specac, Orpington, UK) inside the spectrometer. To obtain difference spectra, 256 scans before and after illumination with two blue LEDs for 2 s (450 nm, 42-70 mW/cm^2, Lumileds, Schiphol, Netherlands) were recorded. The final difference spectrum of LOV was an average of 2048 scans from 8 technical replicates.

For LOV measured in the transmission mode, ~1 µL of the sample with a concentration of 1.7 mM was placed between two BaF_2 windows, sealed with grease and mounted in the spectrometer. Difference spectra were obtained by recording 10,240 scans on 10 technical replicates at a spectral resolution of 2 cm^{-1} and 4 cm^{-1} before and after illumination with a blue LED for 2 s (450 nm, 14 mW/cm^2, Lumileds, Schiphol, Netherlands).

In-Cell Infrared Spectroscopy

The cultivation of HEK-293 cells on a ZnS IRE inside a Bruker IFS66/v spectrometer was achieved by using a home-built cell cultivation chamber. The theoretical penetration depth of the ZnS IRE was calculated according to Equation (S1).[5]

$$d_p = \frac{\lambda_0}{4\pi\sqrt{n_{ZnS}^2 \sin^2\theta - n_{cells}^2}} \quad \text{(Eq. S1)}$$

The ZnS IRE was mounted with a silicon gasket and a Makrolon (polycarbonate) sample chamber on top of an ATR unit inside the spectrometer. A long pass filter with a cut off at 2000 cm^{-1} or 1910 cm^{-1} was placed in front of the MCT detector of the spectrometer. The ATR unit was temperature controlled to 37 °C and optics were purged with dry air. The Makrolon (polycarbonate) sample chamber and IRE were disinfected with ethanol to minimize contaminations. First, 1.5 mL of *Dulbecco´s Modified Eagle´s* medium (DMEM) with 10% fetal calve serum and 1% penicillin-streptomycin was placed on the IRE and an absorbance spectrum was measured with 128 scans using a deuterated triglycine sulfate (DTGS) detector. Then, 1×10^6 cells of the stable cell line of HEK-293 expressing pCRY-PHR in 1 mL DMEM or 5×10^5 HEK-239 cells for transient transfection for LOV expression in 1 mL DMEM were seeded on the IRE. The Makrolon sample chamber was sealed with grease and enclosed by a PVC compartment with a heated cover to prevent condensation of the medium. The PVC compartment was supplied with a constant flow of 3.5 L/h humidified air containing 12% CO_2 by a gas mass controller for a stable neutral pH of the cell culture medium. A concentration of 5% CO_2 was not sufficient, probably because of some leakage of the cell cultivation chamber. Two LEDs were mounted on top of the PVC compartment for later illumination.

Cell growth was followed by measuring absorbance spectra of the cells with 128 scans and a DTGS detector every hour. The stably transfected HEK-293 cells expressing pCRY-PHR were cultivated for 24-25 h in the spectrometer. A spectrum of water vapor was manually subtracted from cell spectra to compensate for vapor artefacts because of long measurement times. Subsequently, a difference spectrum was obtained by recording 1024 scans with a scanner velocity of 150 kHz using an MCT detector before and after illumination of the cells with two blue LEDs (450 nm, 42-70 mW/cm^2, Lumileds, Schiphol, Netherlands) for 40 s. The difference spectrum of pCRY-PHR resulted from 49,152 scans measured on 26 independent preparations. The full recovery of pCRY-

PHR to the dark state within 1 hour was utilized to repeat illumination and measurement for a second time.

The HEK-293 cells for transient transfection were cultivated for 13-14 h. Afterwards 500 µL DMEM medium was added to compensate minor evaporation of the medium over time. HEK-293 cells were transfected with 6 µg pTwistCMV-LOV using 18 µL of a commercial transfection reagent (XtremeGene 9 DNA Transfection Reagent, Roche) according to the protocol of the manufacturer. After 24 h of incubation, intensity spectra with 254 scans and a scanner velocity of 150 kHz using an MCT detector were recorded of the HEK-293 cells expressing LOV before and after illumination with two blue LEDs (450 nm, 42-70 mW/cm^2, Lumileds, Schiphol, Netherlands) for 20 s. 8,448 scans were averaged from 3 biological replicates to obtain the difference spectrum of LOV.

Infrared difference spectra of LOV and pCRY-PHR in *E. coli* BL21 cells were obtained as described previously.[6] For pCRY-PHR 36,864 scans on 18 independent preparations were recorded at a spectral resolution of 4 cm^{-1}. For LOV, 10,240 scans on 10 technical replications were averaged at a spectral resolution of 2 cm^{-1}.

Predictions of Posttranslational Modifications

Posttranslational modifications in LOV were predicted with PTMGPT2[7] and the following I264V-modified FASTA sequence (mutation highlighted in red):

```
>PTAUREO1A LOV DOMAIN
MGSSHHHHHHSSGLVPRGSHMDFSFIKALQTAQQNFVVTDPSLPDNPVVYASQGFLNLTGYSLDQILGRNCRFLQGPE
TDPKAVERIRKAIEQGNDMSVCLLNYRVDGTTFWNQFFIAALRDAGGNVTNFVGVQCKVSDQYAATVTKQQEEEEEAA
ANDDED
```

As a result, a phosphorylation of serine, N-linked and O-linked glycosylation were predicted for LOV:

MGSSHHHHHHSSGLVPRGSHMD$_{238}$FSFIKALQTAQQNFVVTDPSLPDNPVVYASQGFLNLTGYSLDQILGRNCRFLQ
GPETDPKAVERIRKAIEQGNDMSVCLLNYRVDGTTFWNQFFIAALRDAGGNVTNFVGVQCKVSDQYAATVTKQQEEE
EEAAANDDED$_{378}$

Quantum Chemical Calculations

The frequencies of the normal modes of flavin in LOV were calculated using density functional theory (DFT). For the starting geometry, the crystal structure of LOV (PDB: 5A8B) was selected. The oxidized FMN was truncated to lumiflavin and only the amino acid side chains of Gln291, Asn319, Asn329 and Gln350 starting at Cβ were used for the calculations. The positions of the amino acid side chains were fixed either completely or at the Cβ atom and geometry optimization of lumiflavin and amino acids was performed. Geometry optimization and normal mode analysis were performed using DFT with B3LYP functional and a 6-311+G(2d,p) basis set as implemented in Gaussian16.[8] A loss of hydrogen bonding to flavin for both fixed amino acids was simulated by rotating the dihedral angle χ_1 of Asn319 and Asn329 by -89° and +175.9°, respectively. A loss of hydrogen bonding of Asn329 to C(4)=O was simulated by rotating Asn329 towards Asn319 before the geometry optimization. Spectra were scaled in frequency by a single factor of 0.98. The line spectrum was broadened with Lorentzians with a full width at half maximum of 14 cm^{-1} as determined for homogenous broadening of flavin bands.[9] Potential energy distributions for the

normal modes of the carbonyl stretching vibrations of lumiflavin were calculated with an home-written algorithm[10] in Matlab R2022b (The Mathworks, Natick, MA) from the internal force constants and amplitudes calculated by Gaussian16. Normal modes with a potential energy contribution of below 2% were not included.

Supporting Figures

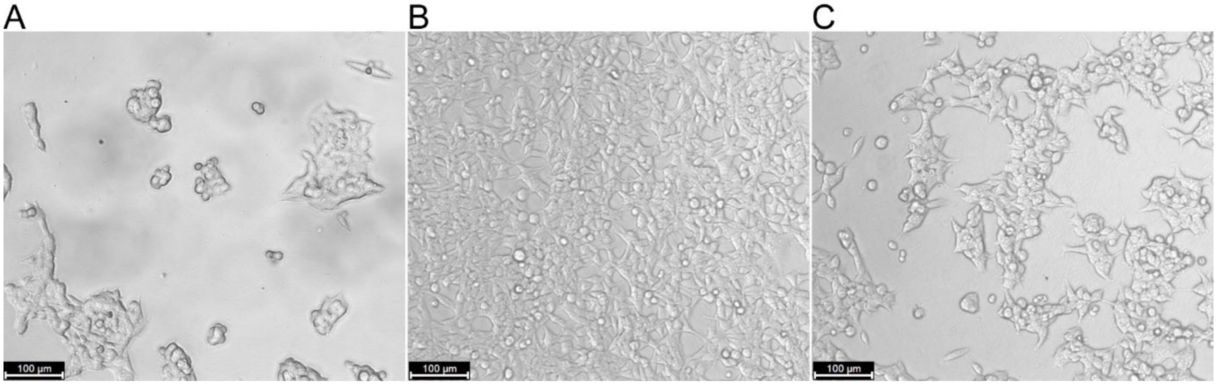

Figure S1. Bright-field images of HEK-293 cells seeded on a T-75 cell culture flask (A), on a ZnS IRE inside a commercial cell culture chamber (B), and on a ZnS IRE inside the spectrometer (C). Pictures were taken 30 h after seeding.

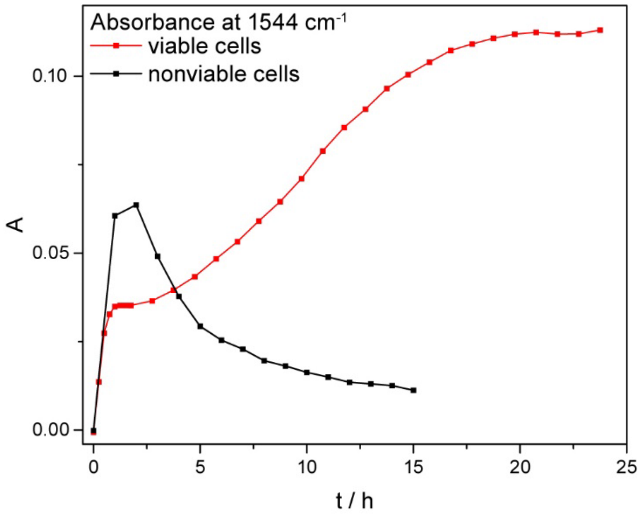

Figure S2. Growth curve of viable and nonviable HEK-293 cells monitored via the amide II band at 1544 cm^{-1}. Viable cells undergo characteristic stages of cell growth, whereas nonviable cells detach from the IRE and show a rapid decrease in absorption of the amide II band.

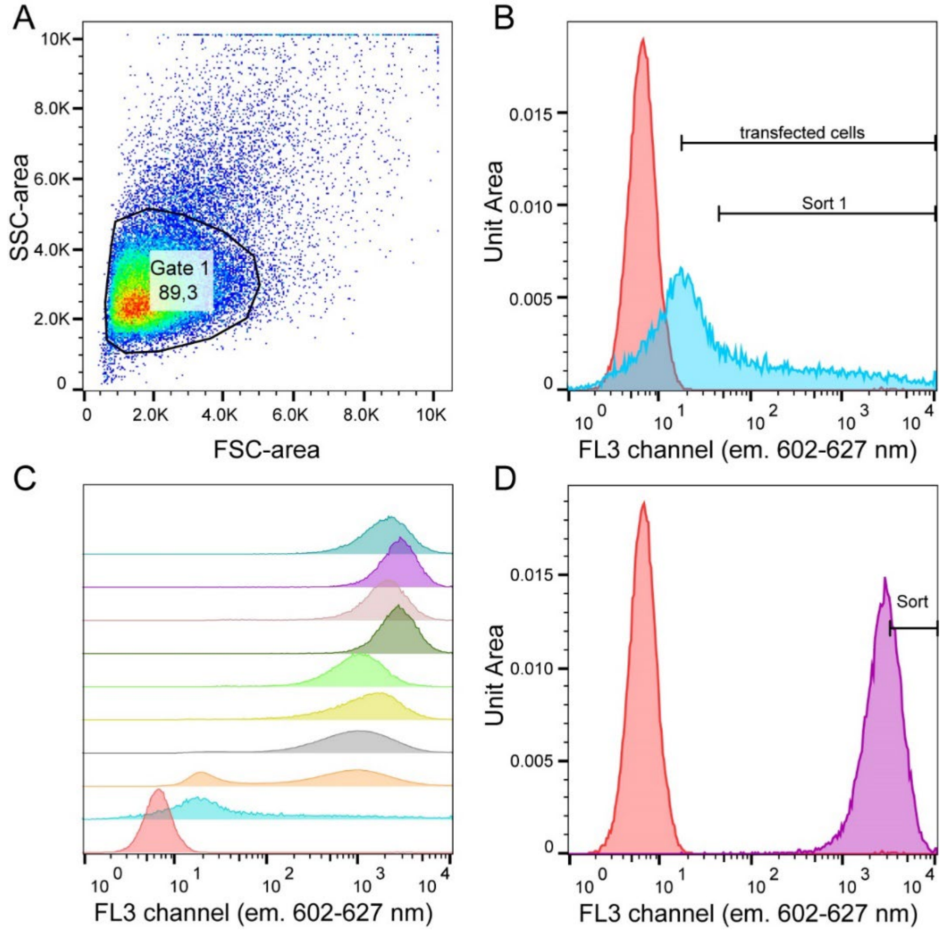

	Sample name	No. days post-transfection	Mean fluorescence intensity FL3 channel	Percentage of population in gate *transfected cells*	Percentage of population in shown sorting gates
	HEK-293 nontransfected	n/a	28.3	0.98	
	HEK-293 pCRY-PHR Sort1	2	406	65.0	37.9 in Sort 1
	HEK-293 pCRY-PHR Sort2	11	880	92.6	
	HEK-293 pCRY-PHR Sort3	21	1236	99.6	
	HEK-293 pCRY-PHR Sort4	70	1548	99.6	
	HEK-293 pCRY-PHR Sort5	82	1148	99.9	
	HEK-293 pCRY-PHR Sort6	87	2895	99.9	
	HEK-293 pCRY-PHR Sort7	102	2122	99.8	
	HEK-293 pCRY-PHR Sort8	118	2933	99.9	44.1 in Sort
	HEK-293 pCRY-PHR Sort9	161	2231	100	

Figure S3. Cell sorting of HEK-293 cells stably expressing pCRY-PHR. (A) Live cells were gated at FSC-area versus SSC-area. (B) The fluorescence in channel FL3 (em. 602 - 627 nm) of live cells was determined in comparison to nontransfected HEK-293 control cells (red). To obtain a stably expressing cell line, the cells with the highest fluorescence intensity were sorted several times during the cultivation period. For example, 37.9% of the population gated as *transfected cells* were sorted in the first sorting two days post transfection (light blue). (C) After three sorting procedures, the fluorescence intensity of the cell population remained stable. (D) For maintenance, the cells with a fluorescence intensity approximately 100 times higher compared to nontransfected control cells were sorted as shown as an example. Figures were created using FlowJo.

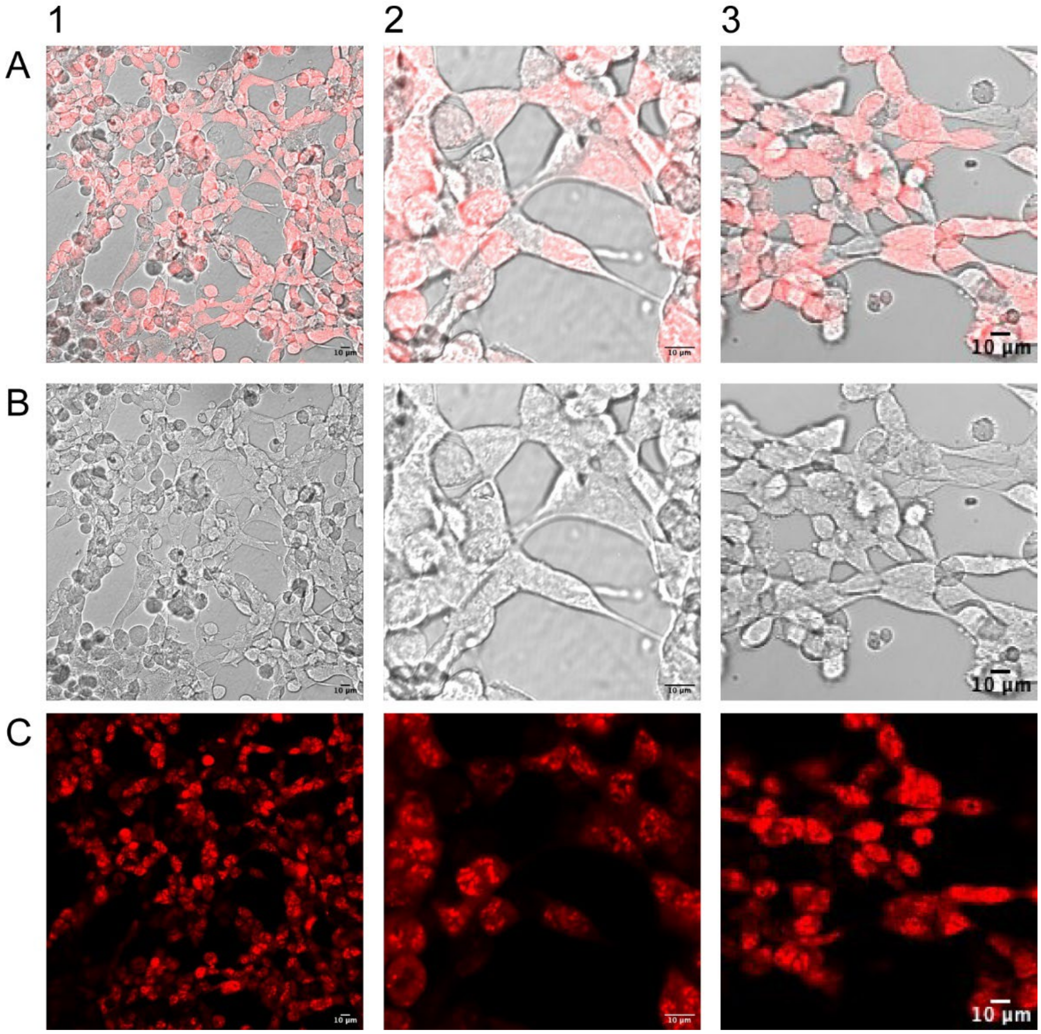

Figure S4. Adherent HEK-293 cells on a ZnS IRE stably expressing pCRY-PHR. The cells were cultured on an ZnS IRE and investigated by confocal imaging (LSM 780, Zeiss). Row A shows overlay pictures composed of bright-field images (row B) and fluorescence signal caused by mCherry (ex. 561 nm, em. 600- 647 nm, row C). If a larger area is considered (column 1), the dense coverage of the IRE with cells is visible, while the zoom on single spindle-shaped cells (columns 2 and 3) indicates high viability.

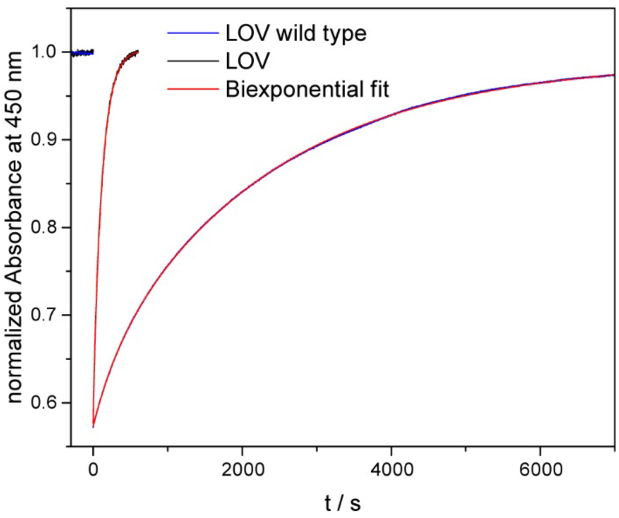

Figure S5. Dark state recovery of LOV-I264V and wild-type LOV at 450 nm and 20 °C. A biexponential fit yielded time constants of τ_1 = 119 (±0.5) s (95%) and τ_2 = 10 (±0.6) s (5%) for LOV and 2290 s (86%) and 320 s (14%) for wild-type LOV. The mutation I264V accelerates the recovery to the dark form by a factor of up to 20 compared to the wild type. Kinetics of wild-type LOV were taken from Herman et al.[11]

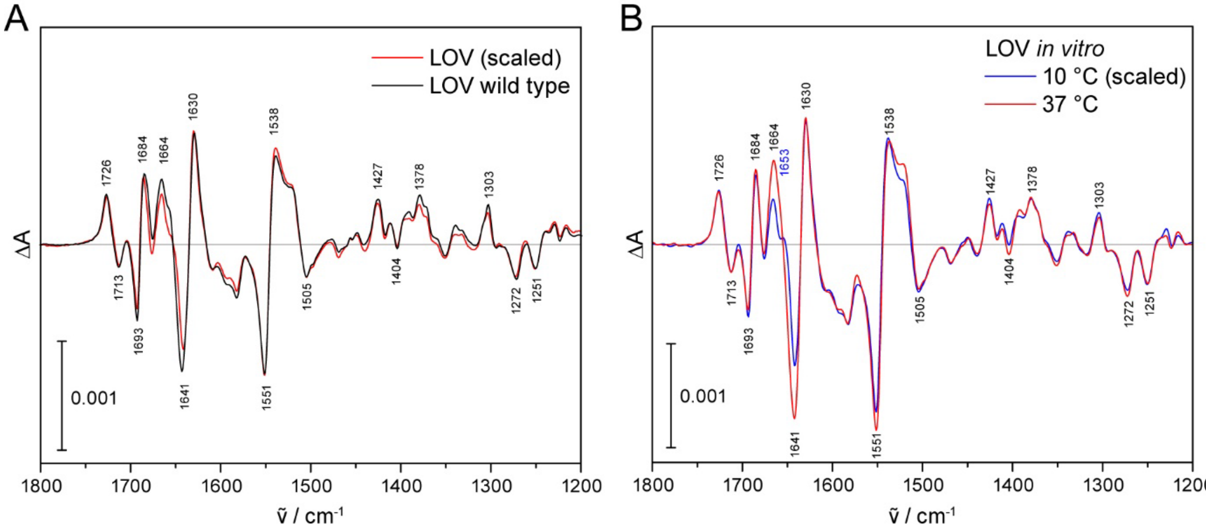

Figure S6. Comparison of the light-induced FTIR difference spectra *in vitro* of wild-type LOV with LOV-I264V, and of LOV-I264V at 10 °C with LOV-I264V at 37 °C. (A) The mutation I264V in LOV has nearly no impact on the light-induced protein response, as observed by comparison to wild-type LOV. The spectrum of wild-type LOV was taken from Herman et al.[12] (B) The signals of the cysteine adduct at 1726 (+) cm^{-1} and the oxidized flavin at 1713 (-) cm^{-1} and 1693 (-) cm^{-1} are insensitive to temperature between 10 °C and 37 °C. Differences at 1653 (-) cm^{-1} are observed, probably because of an already unfolded A´α helix in the dark state and a more pronounced response of the Jα helix of LOV at 37 °C.[12]

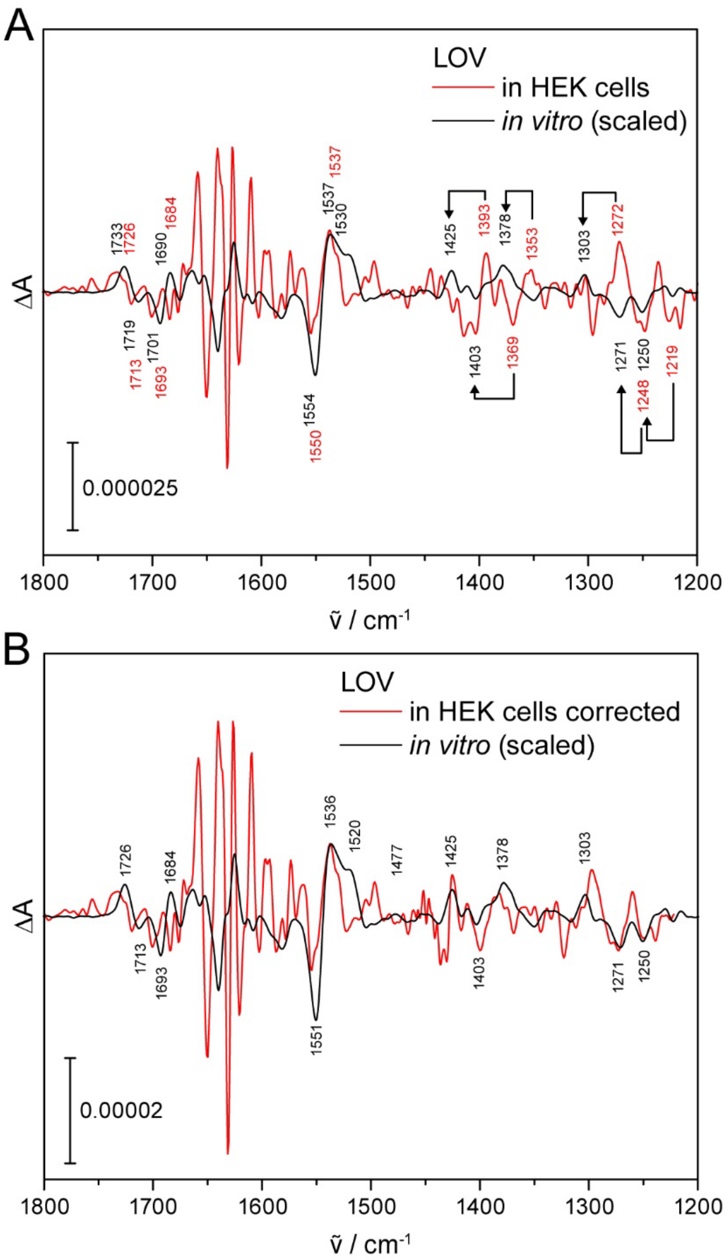

Figure S7. Light-induced ATR FTIR difference spectrum of LOV-I264V in transiently transfected HEK-293 cells compared to *in vitro* before and after correction for anomalous dispersion. (A) Signals in the region of 1450 cm^{-1} to 1200 cm^{-1} in the difference spectrum of LOV-I264V are shifted to lower wavenumber in HEK-293 cells than *in vitro* (black arrows). Spectra were corrected in intensity for the wavelength dependence of the penetration depth. (B) Signals in the difference spectrum of LOV in HEK-293 cells were corrected for the downshift in wavenumbers in the region 1450-1200 cm^{-1} caused by anomalous dispersion by using a scaling function calculated from the shift of characteristic signals shown in (A). In addition, the difference absorbance was scaled in intensity according to the higher penetration depth than *in vitro* resulting from the different refractive indices of water and HEK-293 cells.

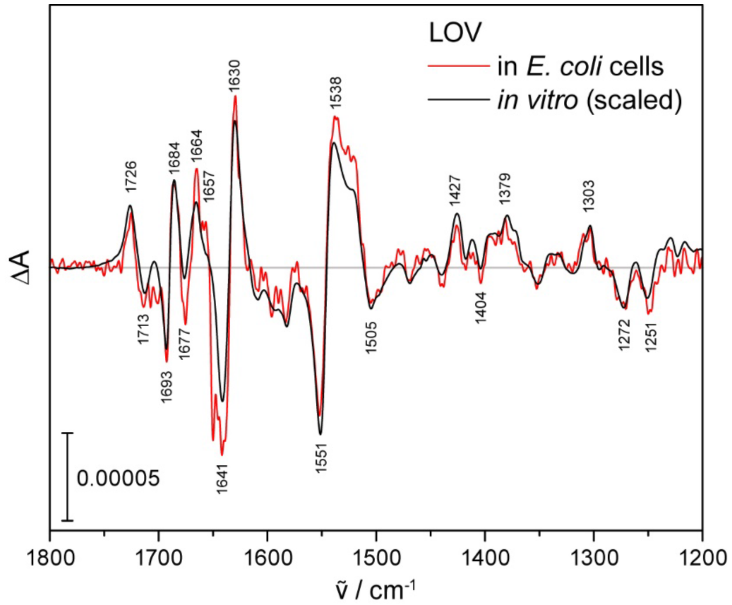

Figure S8. Comparison of the light-induced FTIR difference spectra of LOV in *E. coli* cells and LOV *in vitro*. Spectra of LOV *in vitro* and in *E. coli* cells measured in the transmission mode with a spectral resolution of 2 cm^{-1} are nearly congruent.

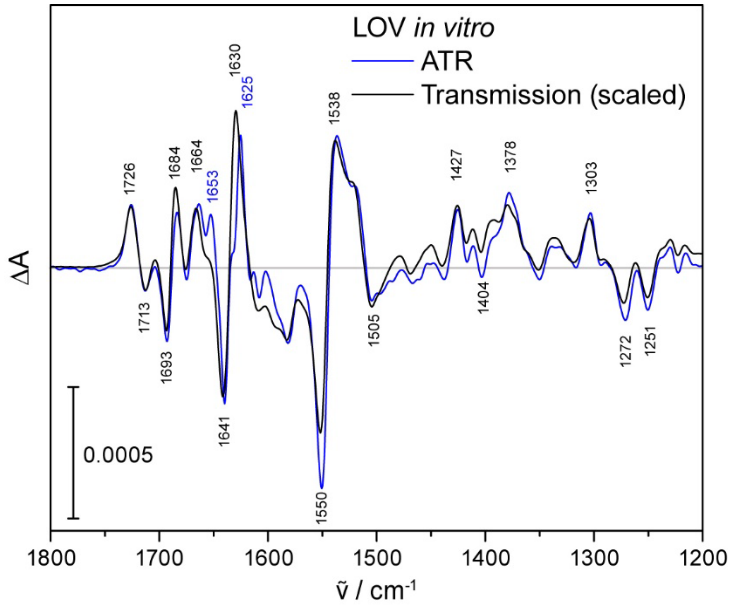

Figure S9. Comparison of the light-induced FTIR difference spectra of LOV *in vitro* recorded with the ATR approach on the same IRE as the HEK-293 cells and LOV *in vitro* recorded in the transmission mode. Spectra obtained by different techniques with a spectral resolution of 4 cm^{-1} showed only minor deviations in the light-induced difference signals of LOV. The signals of the oxidized flavin and the cysteine adduct at 1726 (+), 1713 (-) and 1693 (-) cm^{-1} are not affected by the ATR approach. Deviations are present at 1625 cm^{-1}, which are attributed to the effect of anomalous dispersion at high absorption. The spectrum obtained with the ATR approach was corrected in intensity for the wavelength dependence of the penetration depth.

Table S1. Comparison of the carbonyl signals of the cofactor flavin C(4)=O in different LOV domains in the dark and light state.

Identity	Abbreviation	Oxidized flavin $\tilde{\nu}$ / cm^{-1}	Cysteine adduct $\tilde{\nu}$ / cm^{-1}	Reference
LOV-I264V of aureochrome1a from *Phaeodactylum tricornutum* in HEK-293 cells	*Pt*Aureo1a LOV I264V in HEK	1719	1733	This work
Vivid (VVD-36) from *Neurospora crassa in vitro*	Vivid	1711	1725	[13]
neo1-LOV2 from *Adiantum capillus-veneris in vitro*	Neo1-LOV2	1710	1727	[14]
LovK from *Caulobacter crescentus in vitro*	LovK	1716	1726	[15]
DsLOV-M49S from *Dinoroseobacter shibae in vitro*	*Ds*LOV-M49S	1710	1724	[16]
YtvA from *Bacillus subtilis in vitro*	YtvA	1718	1728	[17]
LOV of aureochrome1a from *Phaeodactylum tricornutum in vitro*	*Pt*Aureo1a LOV	1713	1727	[12]
LOV1-C57S-LOV2 of phototropin from *Chlamydomonas reinhardtii in vitro*	*Cr*Phot LOV2	1718	1730	[18]
LOV2 of phototropin 2 from *Arabidopsis thaliana in vitro*	*At*Phot2 LOV2	1714	1732	[19]
LOV1 of phototropin 2 from *Chlamydomonas reinhardtii in vitro*	*Cr*Phot LOV1	1711	1724	[6]
LOV1 of phototropin 2 from *Chlamydomonas reinhardtii* in *E. coli*	*Cr*Phot LOV1 in *E. coli*	1711	1724	[6]
EL222 from *Erythrobacter litoralis in vitro*	EL222-LOV	1714	1727	[20]
neo1-LOV1 from *Adiantum capillus-veneris in vitro*	Neo1-LOV1	1713	1724	[21]
LOV-I264V of aureochrome1a from *Phaeodactylum tricornutum* in *E. coli*	*Pt*Aureo1a LOV I264V in *E. coli*	1714	1725	This work
LOV-I264V of aureochrome1a from *Phaeodactylum tricornutum in vitro*	*Pt*Aureo1a LOV I264V	1714	1726	This work
LOV of FKF1 from *Arabidopsis thaliana in vitro*	FKF1	1715	1725	[22]
LOV2 of phototropin 1 from *Arabidopsis thaliana in vitro*	*At*Phot1 LOV2	1712	1730	[23]
LOV2 of phototropin 1 from *Avena sativa in vitro*	*As*Phot1 LOV2	1712	1731	[24]
neo1-LOV2-Q1029L from *Adiantum capillus-veneris in vitro*	Neo1-LOV2 Q1029L	1723	1730	[25]

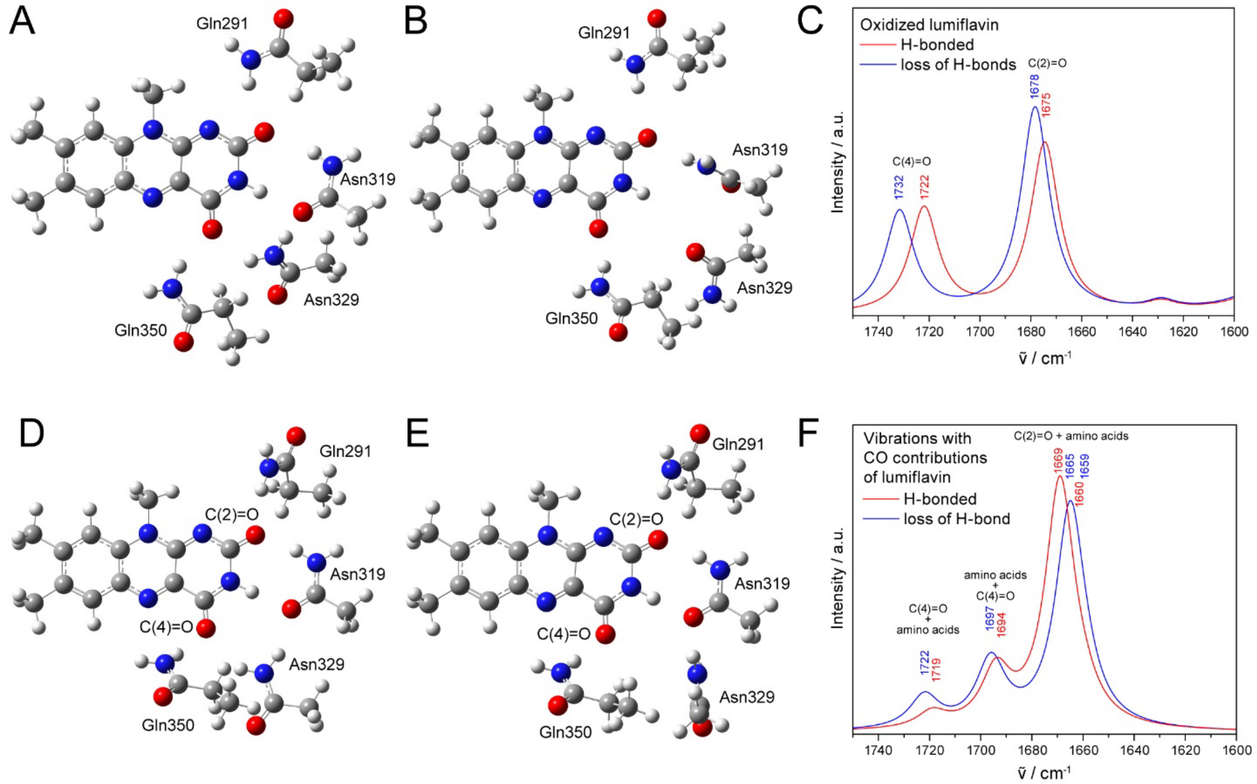

Figure S10. Optimized structures and calculated IR spectra of the oxidized lumiflavin with surrounding amino acids from LOV in the dark. (A) The optimized structure of lumiflavin is based on the crystal structure of LOV (PDB: 5A8B). Surrounding amino acids were kept fixed for geometry optimization. (B) Disruption of hydrogen bonding of Asn329 to C(4)=O and a decrease in hydrogen bonding of Asn319 to C(2)=O was simulated by rotating the corresponding asparagine residues. (C) In the calculated IR spectra, a loss of hydrogen bonding of Asn329 and C(4)=O and a decrease in hydrogen bonding of Asn319 and C(2)=O results in a shift of +10 cm^{-1} and +4 cm^{-1} in the C(4)=O and C(2)=O stretching modes, respectively. Vibrational coupling of the amino acid residues with flavin was not considered in the DFT calculation. The calculated spectrum lacks a carbonyl signal at around 1693 cm^{-1} and overestimates the frequency shift compared to experimental data. (D) In a second approach, lumiflavin and the amino acid residues were included in the geometry optimization, only Cβ of the amino acids was fixed. (E) A loss of hydrogen bonding of Asn329 to C(4)=O of flavin was simulated by rotating Asn329 before geometry optimization. (F) The calculated spectrum of oxidized lumiflavin with surrounding amino acids exhibits three signals for the carbonyl stretching modes of oxidized lumiflavin. As a result of coupling of the amino acids with the carbonyls of lumiflavin, the C(4)=O signal shows two contributions at 1719 cm^{-1} and at 1694 cm^{-1} according to the potential energy distribution for the normal modes of the carbonyl stretching vibrations (Table S2). The loss of hydrogen bonding of Asn329 to C(4)=O results in a shift of +3 cm^{-1} for both C(4)=O stretching modes, which is in agreement with experimental data. However, the loss of a single hydrogen bond is not sufficient to explain the full extent of the shift of +7 cm^{-1} in the experiments.

Table S2. Potential energy distributions for the calculated normal modes of carbonyl stretching modes of lumiflavin with surrounding amino acids. The three most prominent contributions were selected.

Carbonyl stretching modes of lumiflavin with H-bond of Asn329 to C(4)=O				
ṽ / cm^{-1}	Intensity	1.	2.	3.
1719	130	76% flavin ν(C4O)	4% Asn329 ν(CO)	3% Gln350 ν(CO)
1694	456	75% Asn329 ν(CO) & ν(CN)	5% flavin ν(C4O)	3% Asn319 ν(CO)
1669	2037	39% Asn319 ν(CO) & ν(CN)	26% flavin ν(C2O)	7% Asn329 ν(CO)
1660	123	39% flavin ν(C2O)	19% Asn319 δ(NH2) & δ(CNH)	18% Asn319 ν(CO) & ν(CN)
Carbonyl stretching modes of lumiflavin without H-bond of Asn329 to C(4)=O				
ṽ / cm^{-1}	Intensity	1.	2.	3.
1722	270	84% flavin ν(C4O)	2% Gln350 ν(CO)	2% flavin δ(N3-H)
1697	551	90% Gln350 ν(CO) & ν(CN) & δ(CNH)	2% flavin ν(C4O)	
1665	1815	41% Asn319 ν(CO) & ν(CN) & δ(NH2) & δ(CNH)	38% flavin ν(C2O)	2% flavin δ(N3-H)
1659	119	32% flavin ν(C2O)	23% Asn319 δ(NH_2) & δ(CNH)	21% Asn319 ν(CO) & ν(CN)

Supporting References

(1) Mátés, L.; Chuah, M. K.; Belay, E.; Jerchow, B.; Manoj, N.; Acosta-Sanchez, A.; Grzela, D. P.; Schmitt, A.; Becker, K.; Matrai, J.; Ma, L.; Samara-Kuko, E.; Gysemans, C.; Pryputniewicz, D.; Miskey, C.; Fletcher, B.; VandenDriessche, T.; Ivics, Z.; Izsvák, Z., Molecular evolution of a novel hyperactive *Sleeping Beauty* transposase enables robust stable gene transfer in vertebrates. *Nat. Genet.* **2009,** *41* (6), 753-61.

(2) Izsvák, Z.; Ivics, Z., *Sleeping beauty* transposition: biology and applications for molecular therapy. *Mol. Ther.* **2004,** *9* (2), 147-56.

(3) Shin, Y.; Berry, J.; Pannucci, N.; Haataja, M. P.; Toettcher, J. E.; Brangwynne, C. P., Spatiotemporal Control of Intracellular Phase Transitions Using Light-Activated optoDroplets. *Cell* **2017,** *168* (1-2), 159-171.

(4) Goett-Zink, L.; Toschke, A. L.; Petersen, J.; Mittag, M.; Kottke, T., C-Terminal Extension of a Plant Cryptochrome Dissociates from the β-Sheet of the Flavin-Binding Domain. *J. Phys. Chem. Lett.* **2021,** *12* (23), 5558-5563.

(5) Axelrod, D.; Burghardt, T. P.; Thompson, N. L., Total internal reflection fluorescence. *Annu. Rev. Biophys. Bioeng.* **1984,** *13* 247-268.

(6) Goett-Zink, L.; Klocke, J. L.; Bögeholz, L. A. K.; Kottke, T., In-cell infrared difference spectroscopy of LOV photoreceptors reveals structural responses to light altered in living cells. *J. Biol. Chem.* **2020,** *295* (33), 11729-11741.

(7) Shrestha, P.; Kandel, J.; Tayara, H.; Chong, K. T., Post-translational modification prediction via prompt-based fine-tuning of a GPT-2 model. *Nat. Commun.* **2024,** *15* (1), 6699.

(8) Frisch, M. J.; Trucks, G. W.; Schlegel, H. B.; Scuseria, G. E.; Robb, M. A.; Cheeseman, J. R.; Scalmani, G.; Barone, V.; Petersson, G. A.; Nakatsuji, H.; Li, X.; Caricato, M.; Marenich, A. V.; Bloino, J.; Janesko, B. G.; Gomperts, R.; Mennucci, B.; Hratchian, H. P.; Ortiz, J. V.; Izmaylov, A. F.; Sonnenberg, J. L.; Williams; Ding, F.; Lipparini, F.; Egidi, F.; Goings, J.; Peng, B.; Petrone, A.; Henderson, T.; Ranasinghe, D.; Zakrzewski, V. G.; Gao, J.; Rega, N.; Zheng, G.; Liang, W.; Hada, M.; Ehara, M.; Toyota, K.; Fukuda, R.; Hasegawa, J.; Ishida, M.; Nakajima, T.; Honda, Y.; Kitao, O.; Nakai, H.; Vreven, T.; Throssell, K.; Montgomery Jr., J. A.; Peralta, J. E.; Ogliaro, F.; Bearpark, M. J.; Heyd, J. J.; Brothers, E. N.; Kudin, K. N.; Staroverov, V. N.; Keith, T. A.; Kobayashi, R.; Normand, J.; Raghavachari, K.; Rendell, A. P.; Burant, J. C.; Iyengar, S. S.; Tomasi, J.; Cossi, M.; Millam, J. M.; Klene, M.; Adamo, C.; Cammi, R.; Ochterski, J. W.; Martin, R. L.; Morokuma, K.; Farkas, O.; Foresman, J. B.; Fox, D. J. *Gaussian 16 Rev. C.01*, Wallingford, CT, 2016.

(9) Spexard, M.; Immeln, D.; Thöing, C.; Kottke, T., Infrared spectrum and absorption coefficient of the cofactor flavin in water. *Vib. Spectrosc.* **2011,** *57* (2), 282-287.

(10) Thöing, C.; Pfeifer, A.; Kakorin, S.; Kottke, T., Protonated triplet-excited flavin resolved by step-scan FTIR spectroscopy: implications for photosensory LOV domains. *Phys. Chem. Chem. Phys.* **2013,** *15* (16), 5916-5926.

(11) Herman, E.; Sachse, M.; Kroth, P. G.; Kottke, T., Blue-light-induced unfolding of the Jα helix allows for the dimerization of aureochrome-LOV from the diatom *Phaeodactylum tricornutum*. *Biochemistry* **2013,** *52* (18), 3094-3101.

(12) Herman, E.; Kottke, T., Allosterically regulated unfolding of the A'α helix exposes the dimerization site of the blue-light-sensing aureochrome-LOV domain. *Biochemistry* **2015,** *54* (7), 1484-1492.

(13) Yee, E. F.; Oldemeyer, S.; Böhm, E.; Ganguly, A.; York, D. M.; Kottke, T.; Crane, B. R., Peripheral Methionine Residues Impact Flavin Photoreduction and Protonation in an Engineered LOV Domain Light Sensor. *Biochemistry* **2021,** *60* (15), 1148-1164.

(14) Iwata, T.; Yamamoto, A.; Tokutomi, S.; Kandori, H., Hydration and temperature similarly affect light-induced protein structural changes in the chromophoric domain of phototropin. *Biochemistry* **2007,** *46* (23), 7016-7021.

(15) Alexandre, M. T.; Purcell, E. B.; van Grondelle, R.; Robert, B.; Kennis, J. T.; Crosson, S., Electronic and protein structural dynamics of a photosensory histidine kinase. *Biochemistry* **2010,** *49* (23), 4752-4759.

(16) Maia, R. N. A.; Ehrenberg, D.; Oldemeyer, S.; Knieps-Grünhagen, E.; Krauss, U.; Heberle, J., Real-Time Tracking of Proton Transfer from the Reactive Cysteine to the Flavin Chromophore of a Photosensing Light Oxygen Voltage Protein. *J. Am. Chem. Soc.* **2021,** *143* (32), 12535-12542.
(17) He, Y.; Collado, J. T.; Iuliano, J. N.; Woroniecka, H. A.; Hall, C. R.; Gil, A. A.; Laptenok, S. P.; Greetham, G. M.; Illarionov, B.; Bacher, A.; Fischer, M.; French, J. B.; Lukacs, A.; Meech, S. R.; Tonge, P. J., Elucidating the Signal Transduction Mechanism of the Blue-Light-Regulated Photoreceptor YtvA: From Photoactivation to Downstream Regulation. *ACS Chem. Biol.* **2024,** *19* (3), 696-706.
(18) Pfeifer, A.; Mathes, T.; Lu, Y.; Hegemann, P.; Kottke, T., Blue light induces global and localized conformational changes in the kinase domain of full-length phototropin. *Biochemistry* **2010,** *49* (5), 1024-1032.
(19) Pfeifer, A.; Majerus, T.; Zikihara, K.; Matsuoka, D.; Tokutomi, S.; Heberle, J.; Kottke, T., Time-resolved Fourier transform infrared study on photoadduct formation and secondary structural changes within the phototropin LOV domain. *Biophys. J.* **2009,** *96* (4), 1462-1470.
(20) Chaudhari, A. S.; Chatterjee, A.; Domingos, C. A. O.; Andrikopoulos, P. C.; Liu, Y.; Andersson, I.; Schneider, B.; Lórenz-Fonfria, V. A.; Fuertes, G., Genetically encoded non-canonical amino acids reveal asynchronous dark reversion of chromophore, backbone, and side-chains in EL222. *Protein Sci* **2023,** *32* (4), e4590.
(21) Iwata, T.; Nozaki, D.; Tokutomi, S.; Kandori, H., Comparative investigation of the LOV1 and LOV2 domains in *Adiantum* phytochrome3. *Biochemistry* **2005,** *44* (20), 7427-7434.
(22) Kikuchi, S.; Unno, M.; Zikihara, K.; Tokutomi, S.; Yamauchi, S., Vibrational assignment of the flavin-cysteinyl adduct in a signaling state of the LOV domain in FKF1. *J. Phys. Chem. B* **2009,** *113* (9), 2913-2921.
(23) Yamamoto, A.; Iwata, T.; Sato, Y.; Matsuoka, D.; Tokutomi, S.; Kandori, H., Light signal transduction pathway from flavin chromophore to the Jα helix of *Arabidopsis* phototropin1. *Biophys. J.* **2009,** *96* (7), 2771-2778.
(24) Alexandre, M. T.; van Grondelle, R.; Hellingwerf, K. J.; Kennis, J. T., Conformational heterogeneity and propagation of structural changes in the LOV2/Jα domain from *Avena sativa* phototropin 1 as recorded by temperature-dependent FTIR spectroscopy. *Biophys. J.* **2009,** *97* (1), 238-247.
(25) Nozaki, D.; Iwata, T.; Ishikawa, T.; Todo, T.; Tokutomi, S.; Kandori, H., Role of Gln1029 in the photoactivation processes of the LOV2 domain in *Adiantum* phytochrome3. *Biochemistry* **2004,** *43* (26), 8373-8379.
